# Supplementary material for: Risk communication in tables versus text: a registered report randomized trial on ‘fact boxes'
Source: R Soc Open Sci. 2020 Mar 25;7(3):190876. doi: 10.1098/rsos.190876 (PMC7137953; doi:10.1098/rsos.190876)
Supplement: Supplement [file rsos190876supp1.docx]

**Supplement**

Title: Risk communication in tables vs. text: a Registered Report randomised trial on 'fact boxes' (Brick et al., 2020, *Royal Society Open Science*)

Table S1. *Correlations between key variables at baseline.*

| Pearson's *r*(2303) | *M* | *SD* | 1 | 2 | 3 | 4 | 5 | 6 |
| --- | --- | --- | --- | --- | --- | --- | --- | --- |
| 1 Comprehension | 0.75 | 0.26 |  |  |  |  |  |  |
| 2 Trust | 3.57 | 0.85 | .25 |  |  |  |  |  |
| 3 Engaged | 3.58 | 0.97 | .28 | .48 |  |  |  |  |
| 4 Informed | 5.47 | 1.16 | .34 | .39 | .39 |  |  |  |
| 5 Treatment decision | 0.62 | 0.48 | .10 | .18 | .12 | .24 |  |  |
| 6 Attitude - Vaccination | 3.66 | 0.94 | .21 | .31 | .21 | .26 | .20 |  |
| 7 Attitude - Antibiotics | 3.82 | 0.82 | .27 | .29 | .23 | .27 | .18 | .54 |

*Note.* Correlations .08 and above are *p* < .01. Treatment decision at baseline was coded yes = 1, all other answers = 0.

**
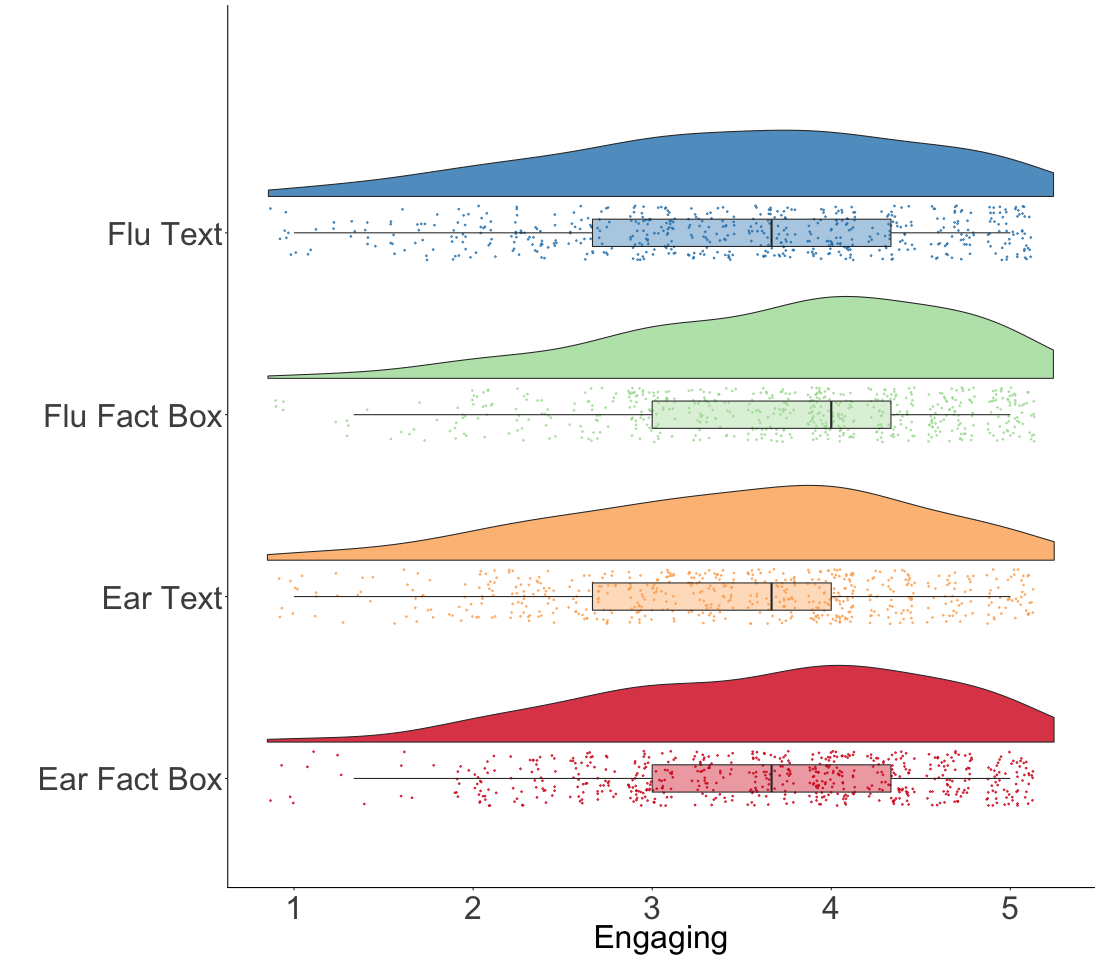
**

*Figure S1.* Ratings of how engaging the material was by experimental condition (baseline).

**
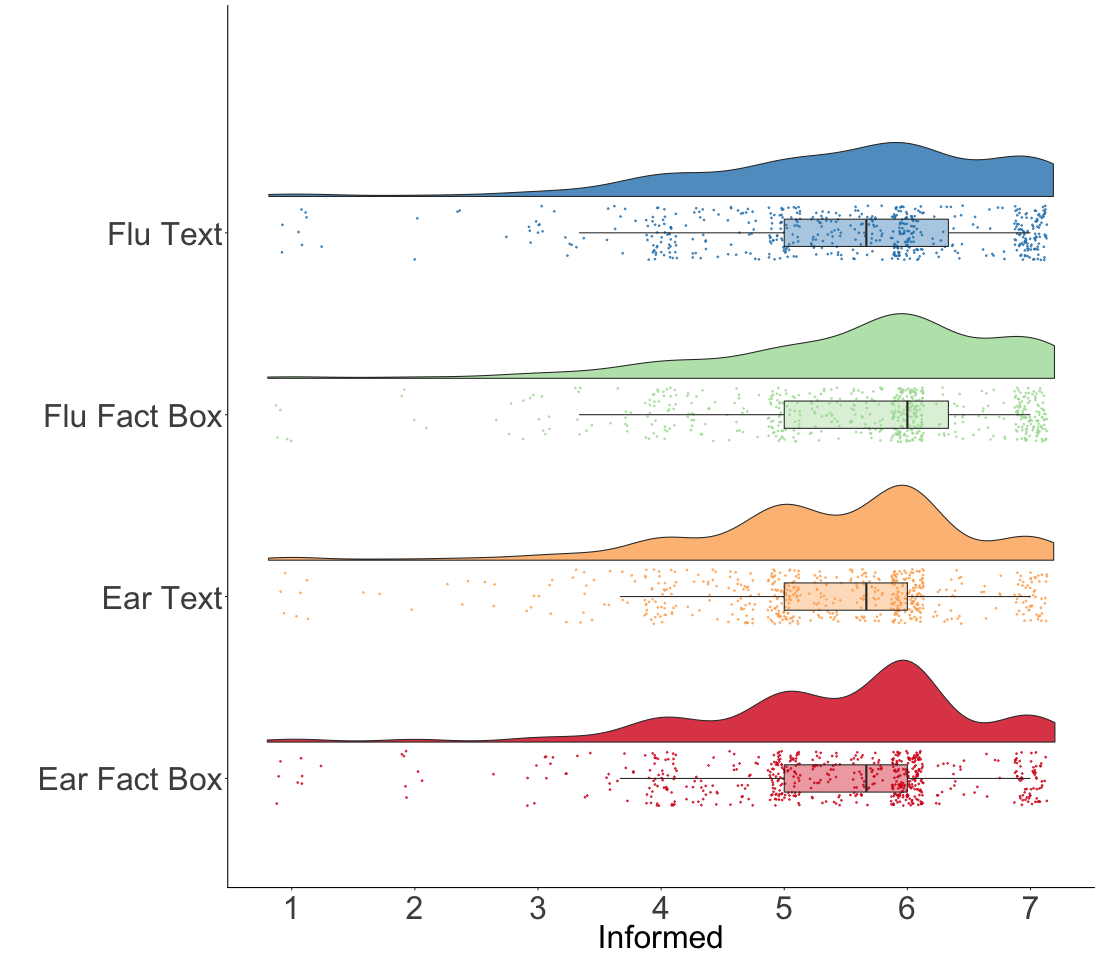
**

*Figure S2.* Ratings of how informed participants felt by experimental condition (baseline).

*
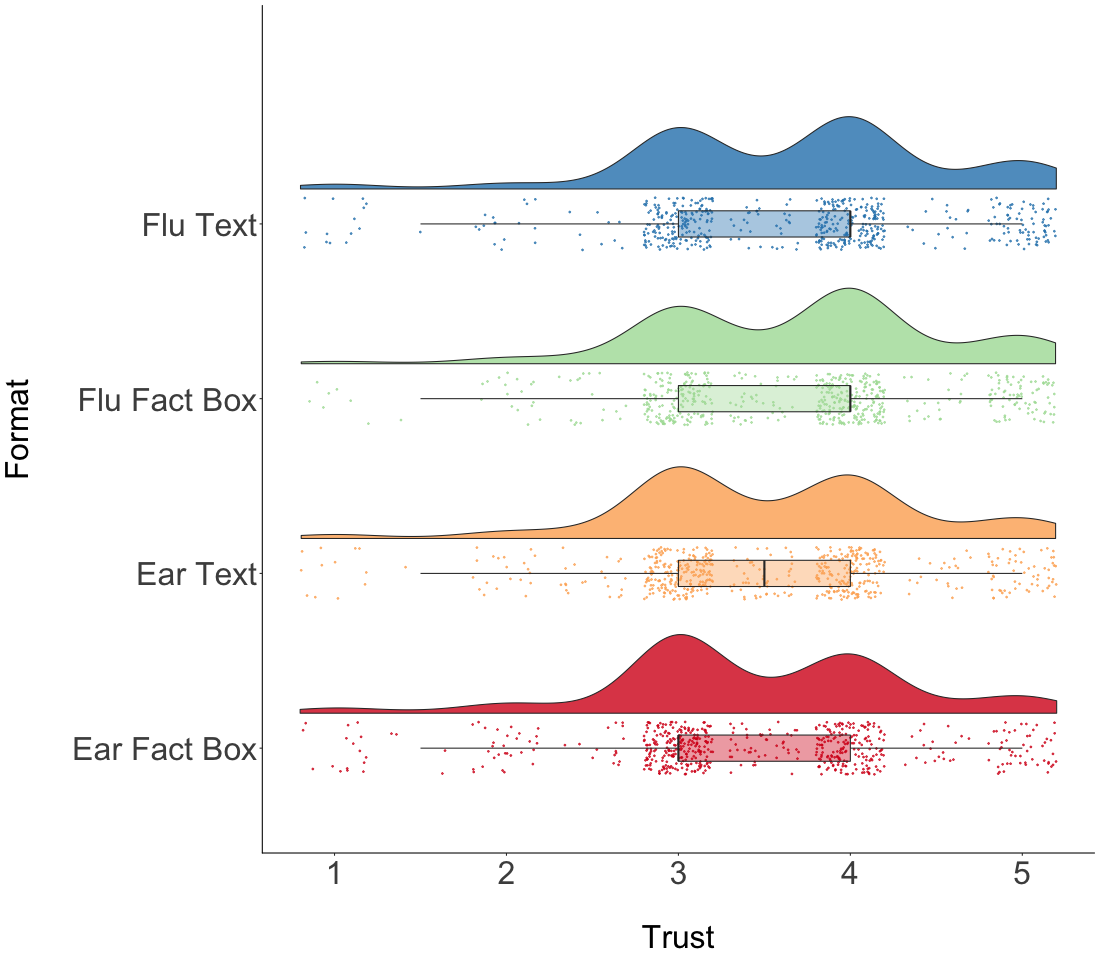
Figure S3.* Ratings of trust in the evidence by experimental condition (baseline).

**
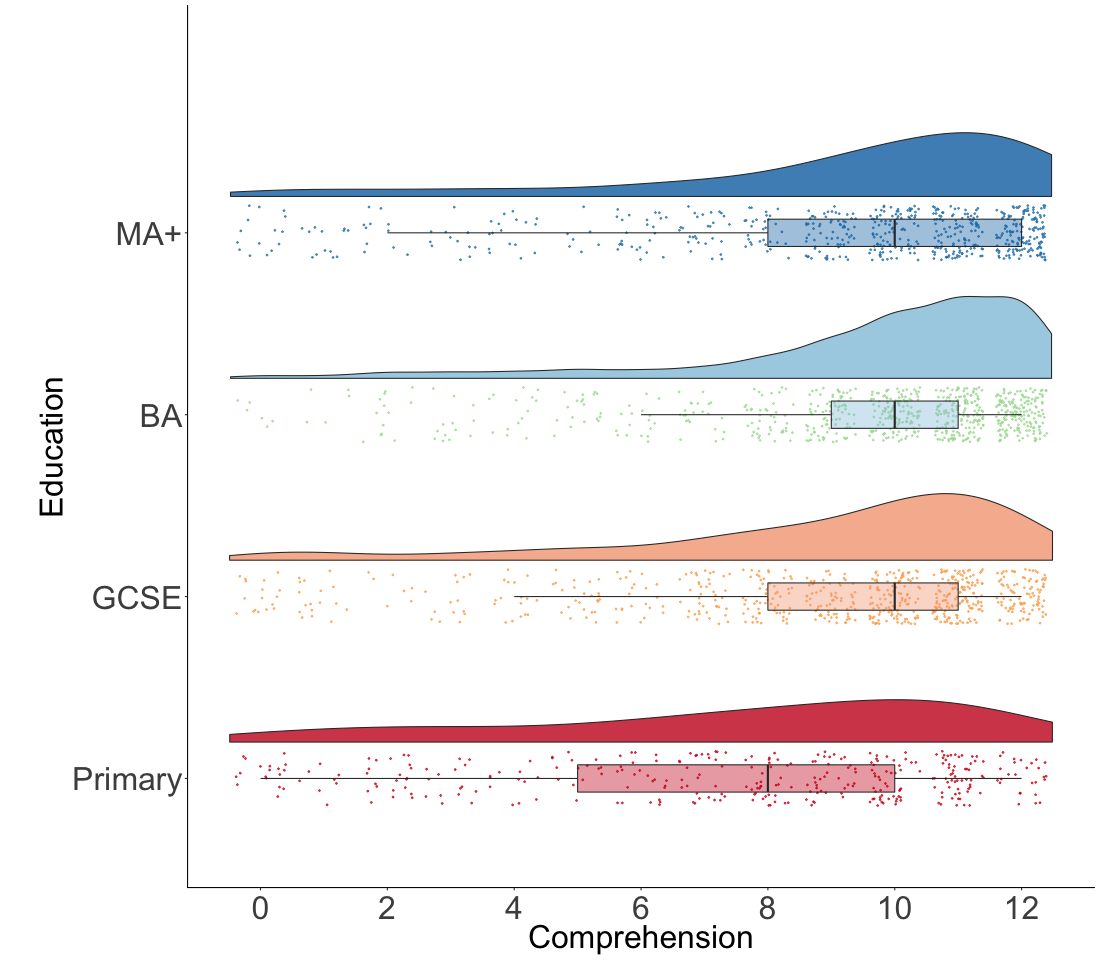
**

*Figure S4.* Baseline comprehension for different levels of educational attainment (baseline).

Table S2. *Treatment decision by format at baseline.*

|  | Fact box | Text |
| --- | --- | --- |
| *n* | *1177* | *1128* |
|  | *Count, (%)* | |
| Yes | 746 (**63.4%**) | 691 (**61.3%**) |
| No | 99 (**8.4%**) | 116 (**10.3%**) |
| No difference | 203 (**17.2%**) | 192 (**17.0%**) |
| Unsure | 129 (**11.0%**) | 129 (**11.4%**) |
|  |  |  |

**
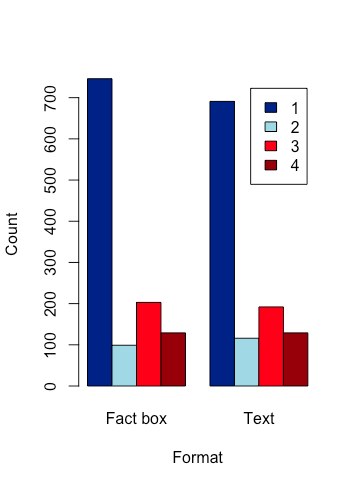
**

*Figure S5a.* Treatment decision by format. 1 = Yes, 2 = No, 3 = No difference, 4 = Unsure.

**
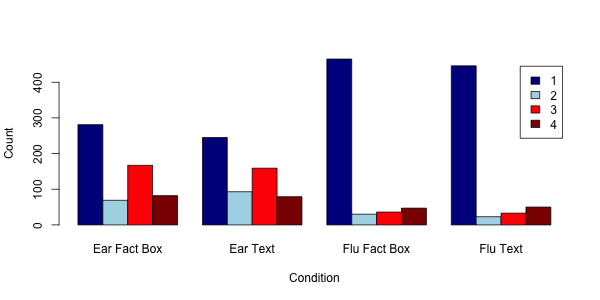
**

*Figure S5b.* Treatment decision by format. 1 = Yes, 2 = No, 3 = No difference, 4 = Unsure.


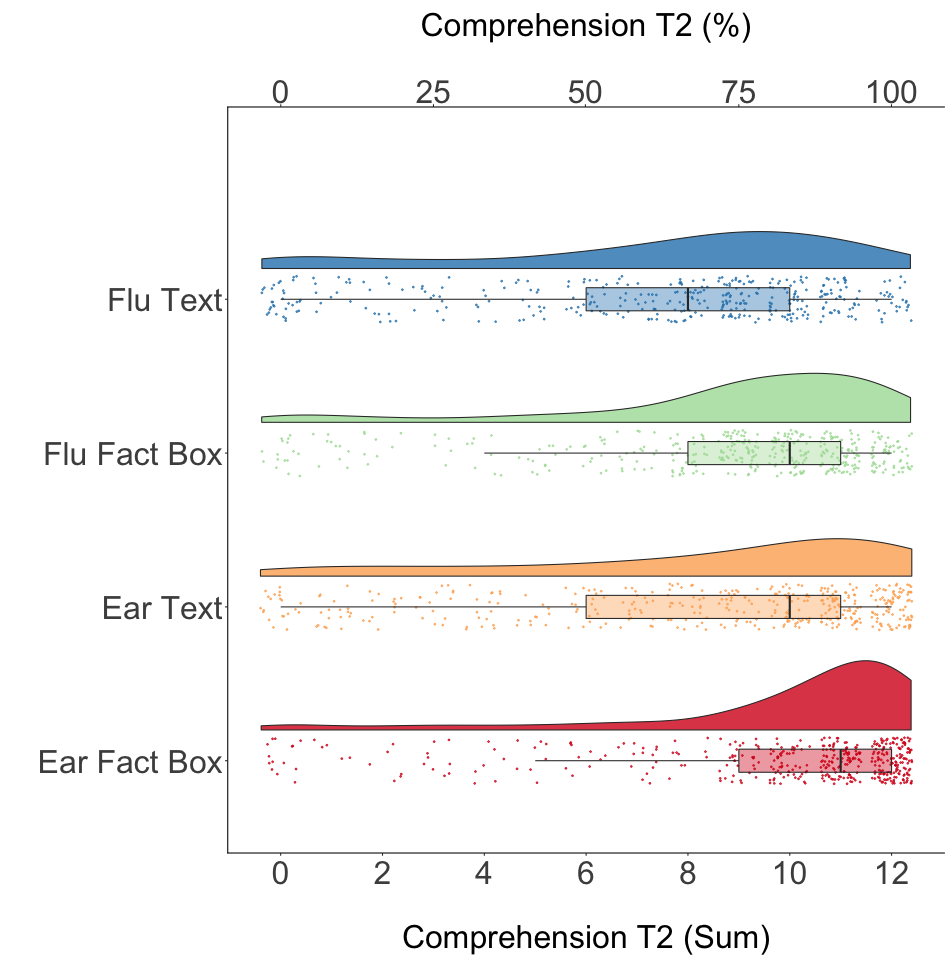


*Figure S6.* Follow-up comprehension by experimental condition (Time 2).

**Comprehension items**

All 12 items from the Ear Fact Box condition are shown. See the questionnaires for the wording for all conditions. Items were shown across several pages and in the order below.

1: {min=0 max=100} Out of 100 children with a middle ear infection who took antibiotics, how many experienced a ruptured eardrum?

9: {min=0 max=100} Out of 100 children with a middle ear infection who took antibiotics, how many experienced an adverse effect (e.g., vomiting, diarrhoea or rash)?

10: {min=0 max=100} Out of 100 children with a middle ear infection who took placebo, how many continued to have impaired hearing 4-6 weeks after diagnosis?

2: Children who took which treatment had a higher chance of developing a ruptured eardrum?

<1> Antibiotics

<2> Both antibiotics and placebo were the same

<3> Placebo

<4> This information is not shown

<5> I don't know

3: Children who took which treatment had a higher chance of experiencing pain 4-7 days after diagnosis?

<1> Antibiotics

<2> Both antibiotics and placebo were the same

<3> Placebo

<4> This information is not shown

<5> I don't know

4: Out of 100 children who received placebo, how many had issues with hearing 4-6 weeks after diagnosis?

<1> Almost none

<2> About a quarter

<3> About half

<4> About three quarters

<5> I don't know

12: Which result was less common in children who took antibiotics compared with those who took a placebo?

<1> Pain

<2> Hearing issues

<3> Adverse effects

<4> This information is not shown

<5> I don't know

5: How did antibiotics affect children's hearing 4-6 weeks after diagnosis compared to placebo?

<1> Antibiotics REDUCED hearing

<2> Antibiotics IMPROVED hearing

<3> The effect was the same in both groups

<4> This information is not shown

<5> I don't know

6: Which of these statements best describes the evidence shown here?

<1> Antibiotics had no effect

<2> Antibiotics only caused harm

<3> Antibiotics only caused benefits

<4> Antibiotics caused both harm and benefits

<5> I don't know

11: Which group was less likely to experience pain 4-7 days after diagnosis?

<1> Children who took antibiotics

<2> Children who took a placebo

<3> The effect was the same in both groups

<4> This information was not shown

<5> I don't know

7: Which group experienced more adverse effects (harms) such as vomiting, diarrhoea or rash?

<1> Children who took antibiotics

<2> Children who took a placebo

<3> The effect was the same in both groups

<4> This information was not shown

<5> I don't know

8: {min=0 max=100} How many more of the 100 children taking antibiotics experienced adverse effects (harms) compared to the 100 children taking placebo? Enter 0 if there was no difference.

**Open Response Comprehension Histograms: Items (1, 8, 9, 10) by Time Point and Format**

*Ear Item 1*

**
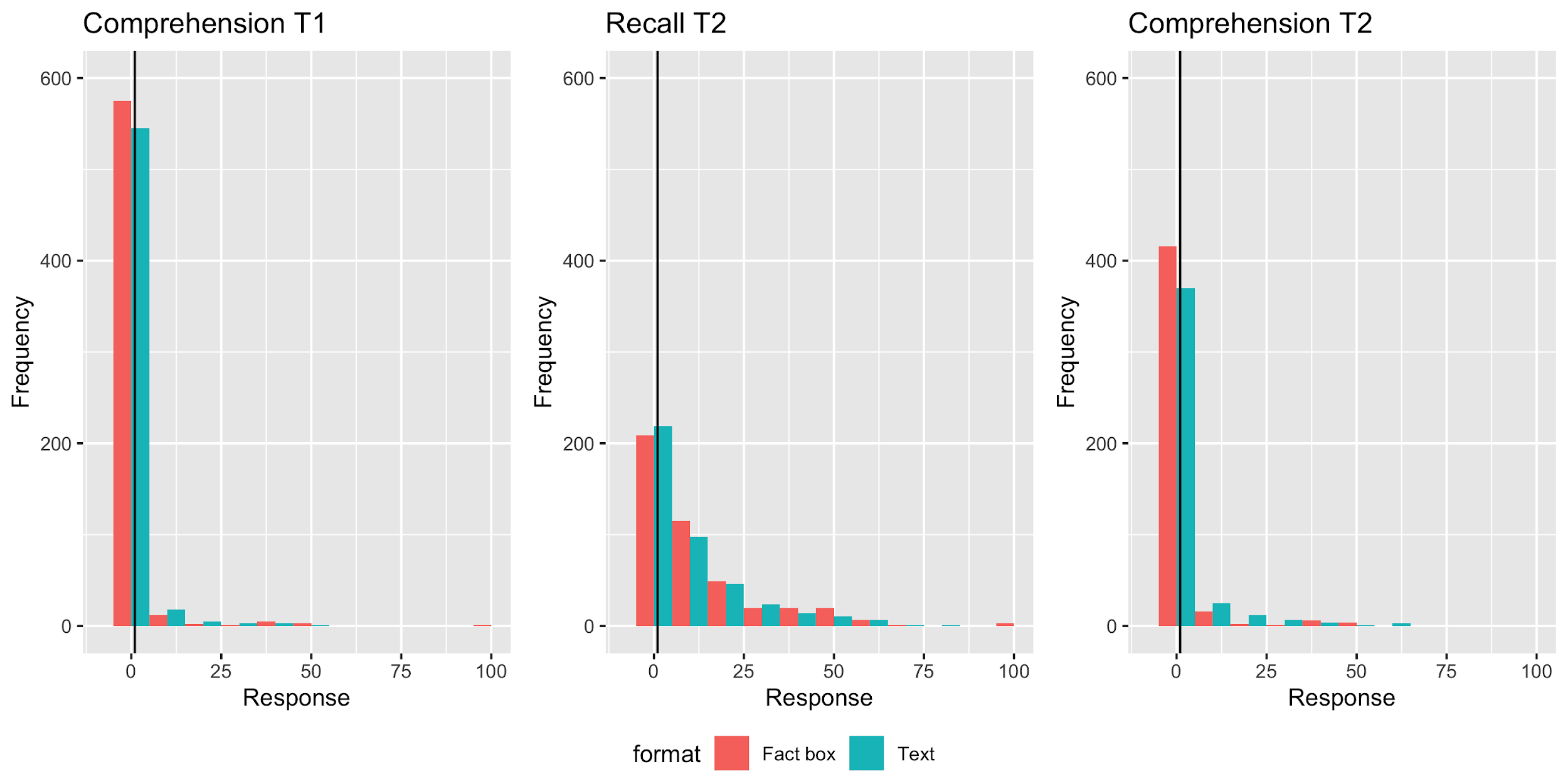
***Ear Item 8***
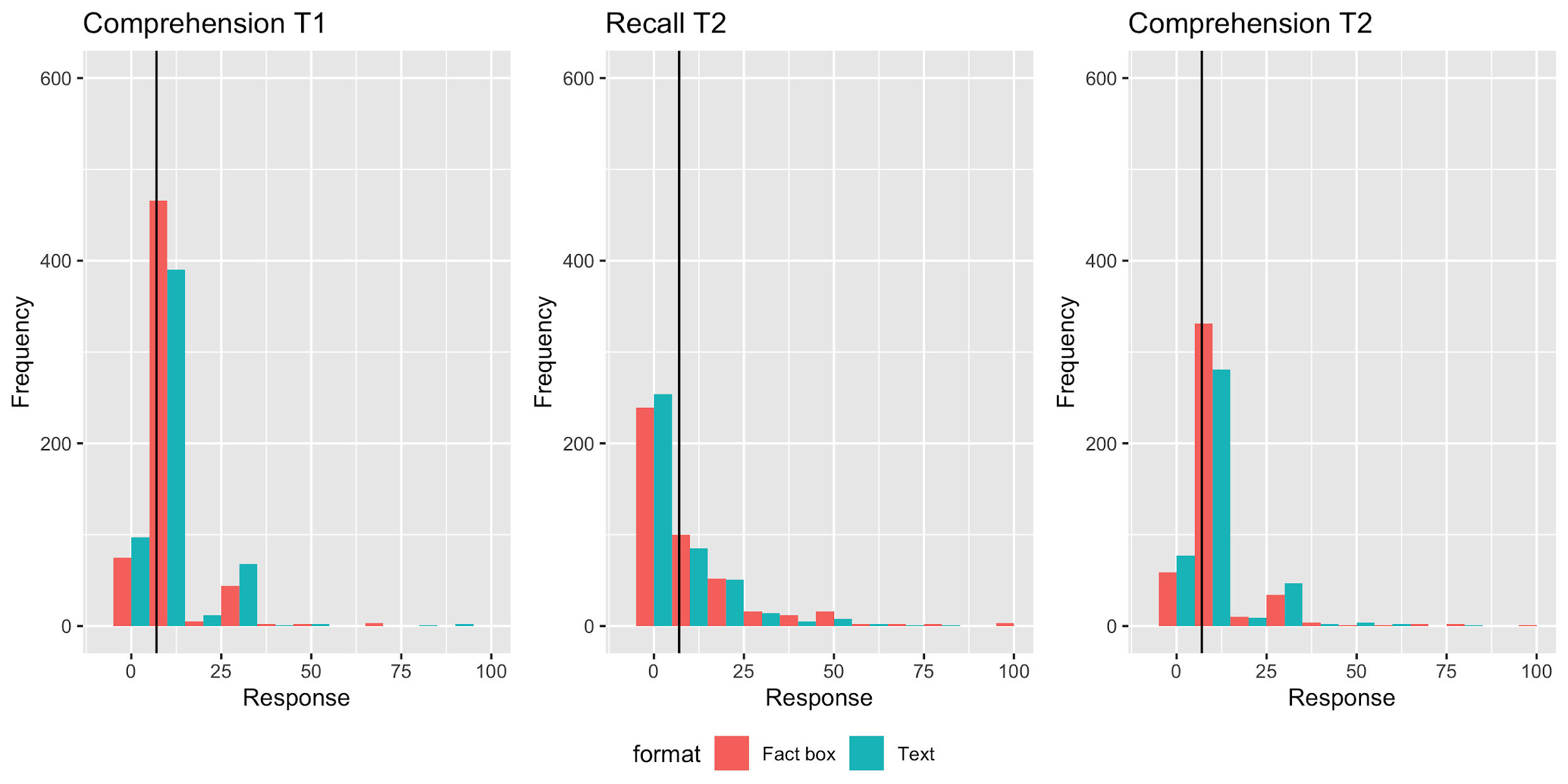
**

*Ear Item 9***
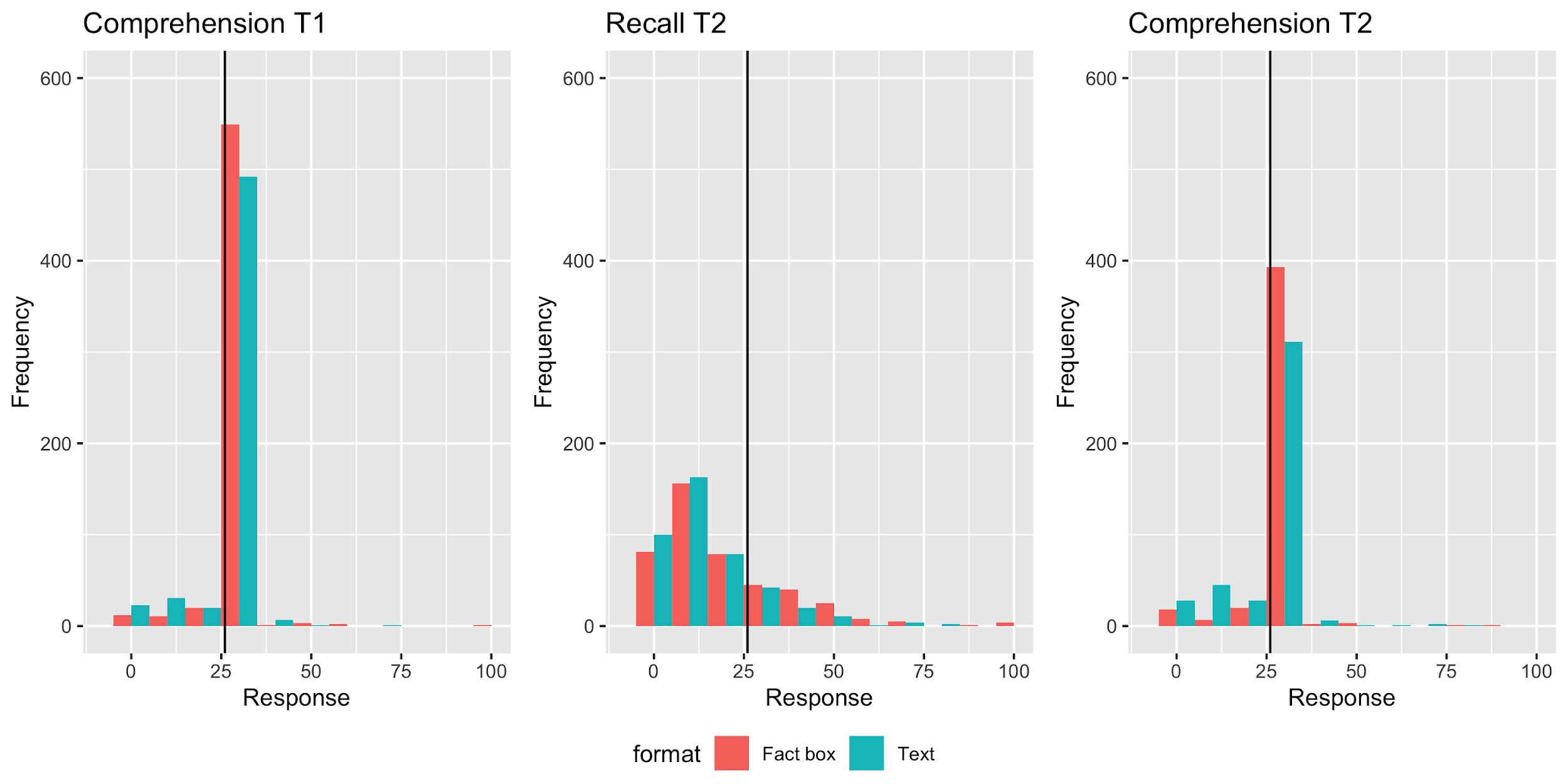
***Ear Item 10***
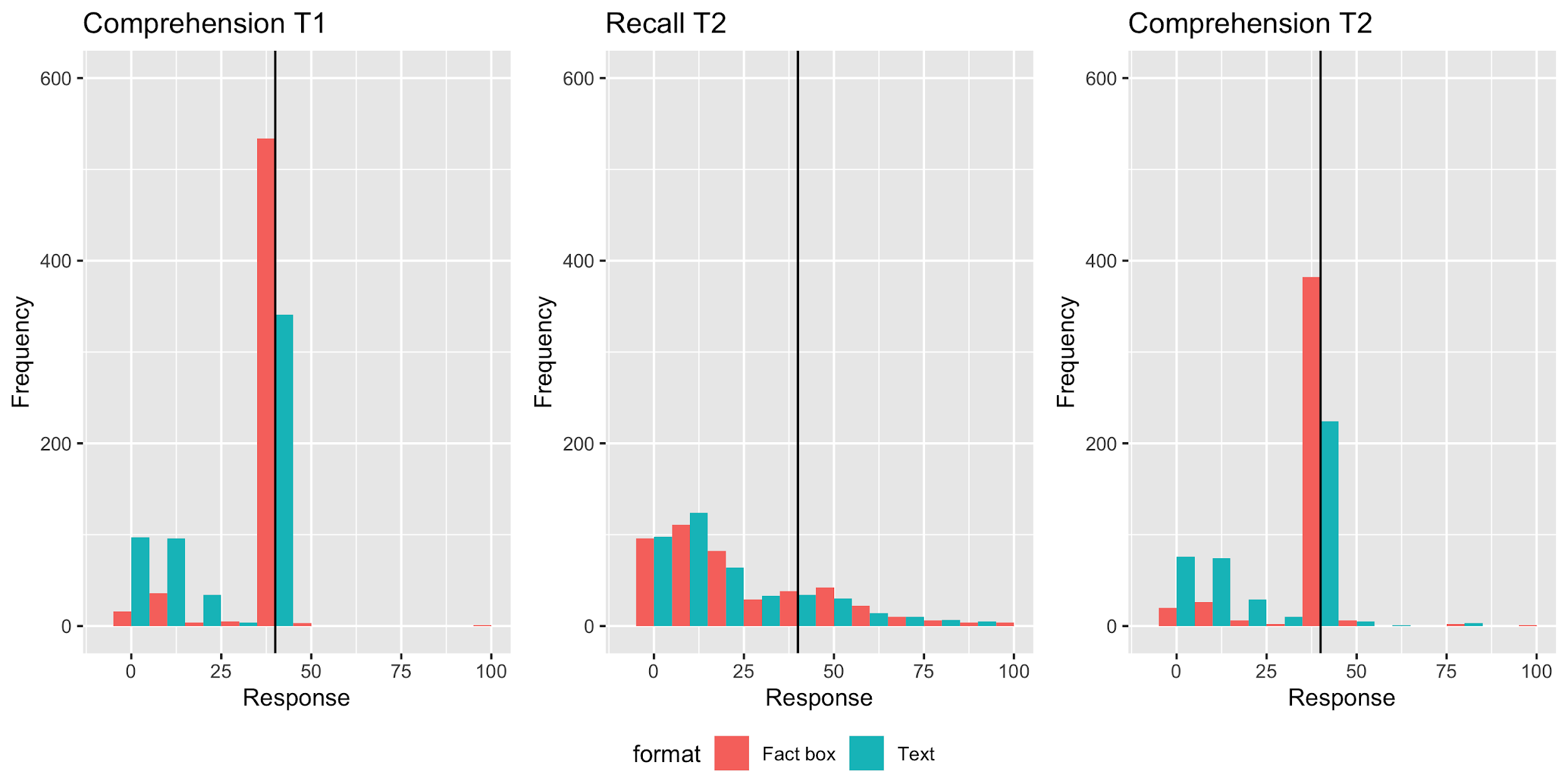
**

*Flu Item 1***
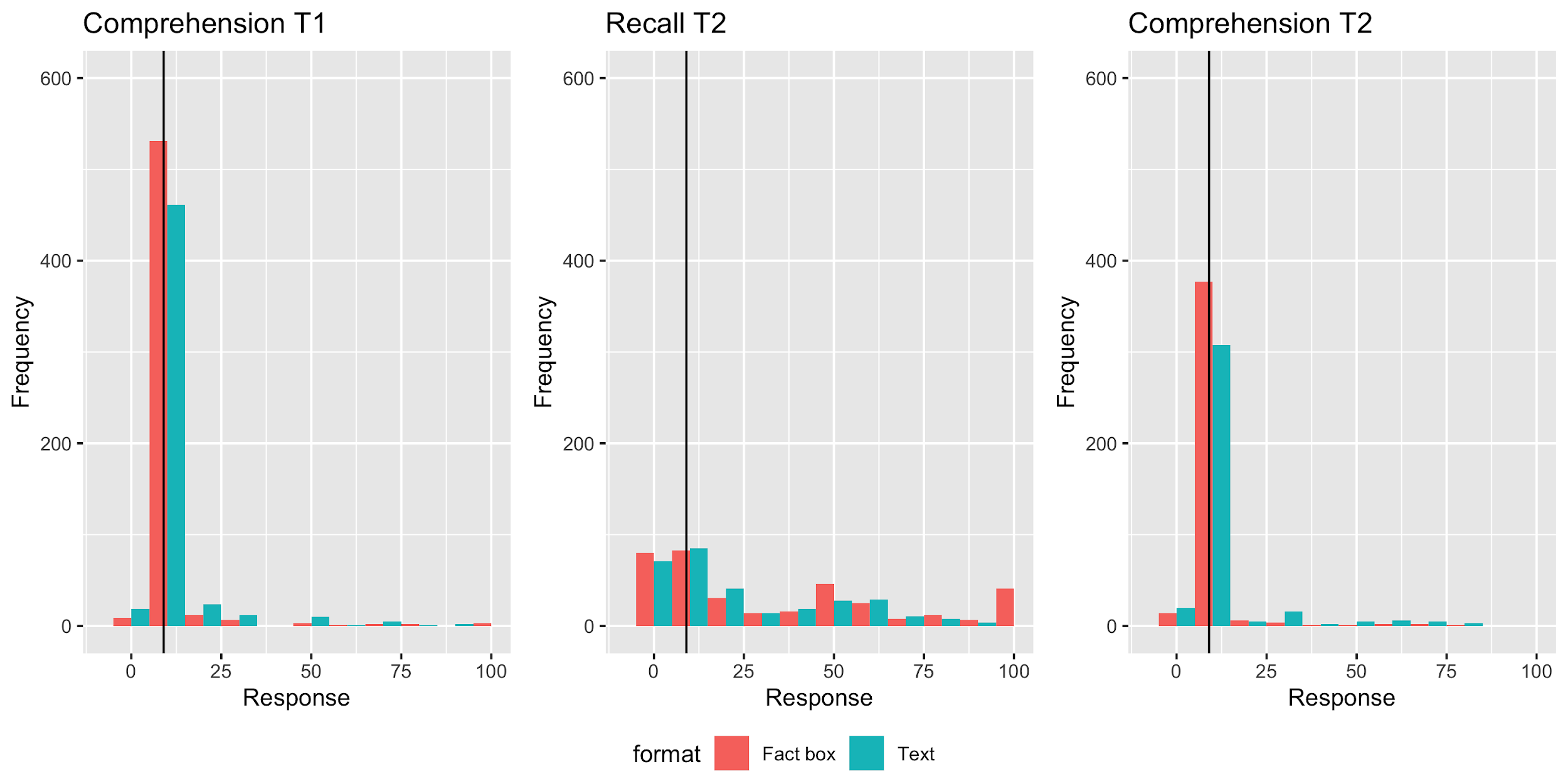
***Flu Item 8* **
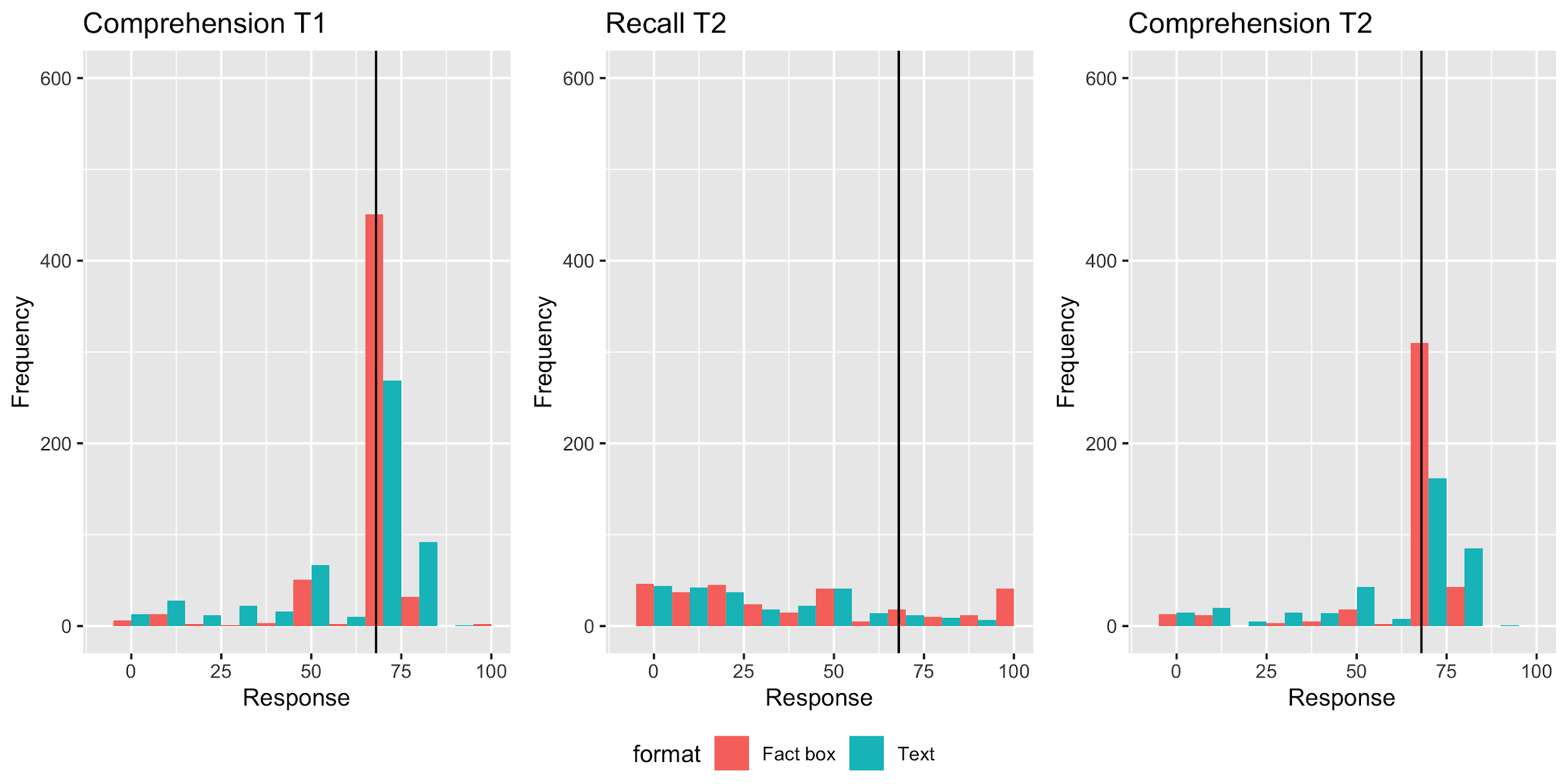
**

*Flu Item 9* **
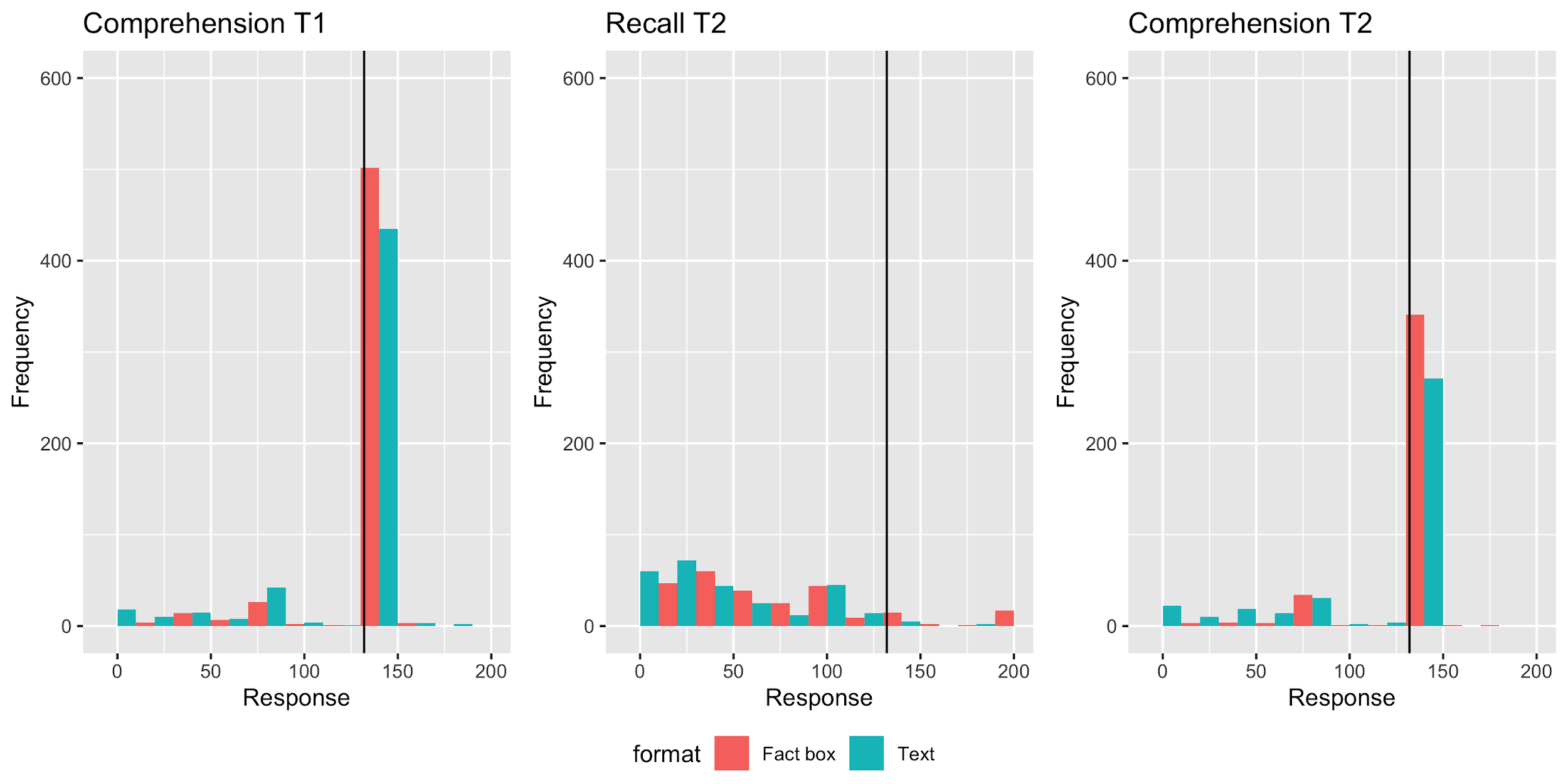
***Flu Item 10* **
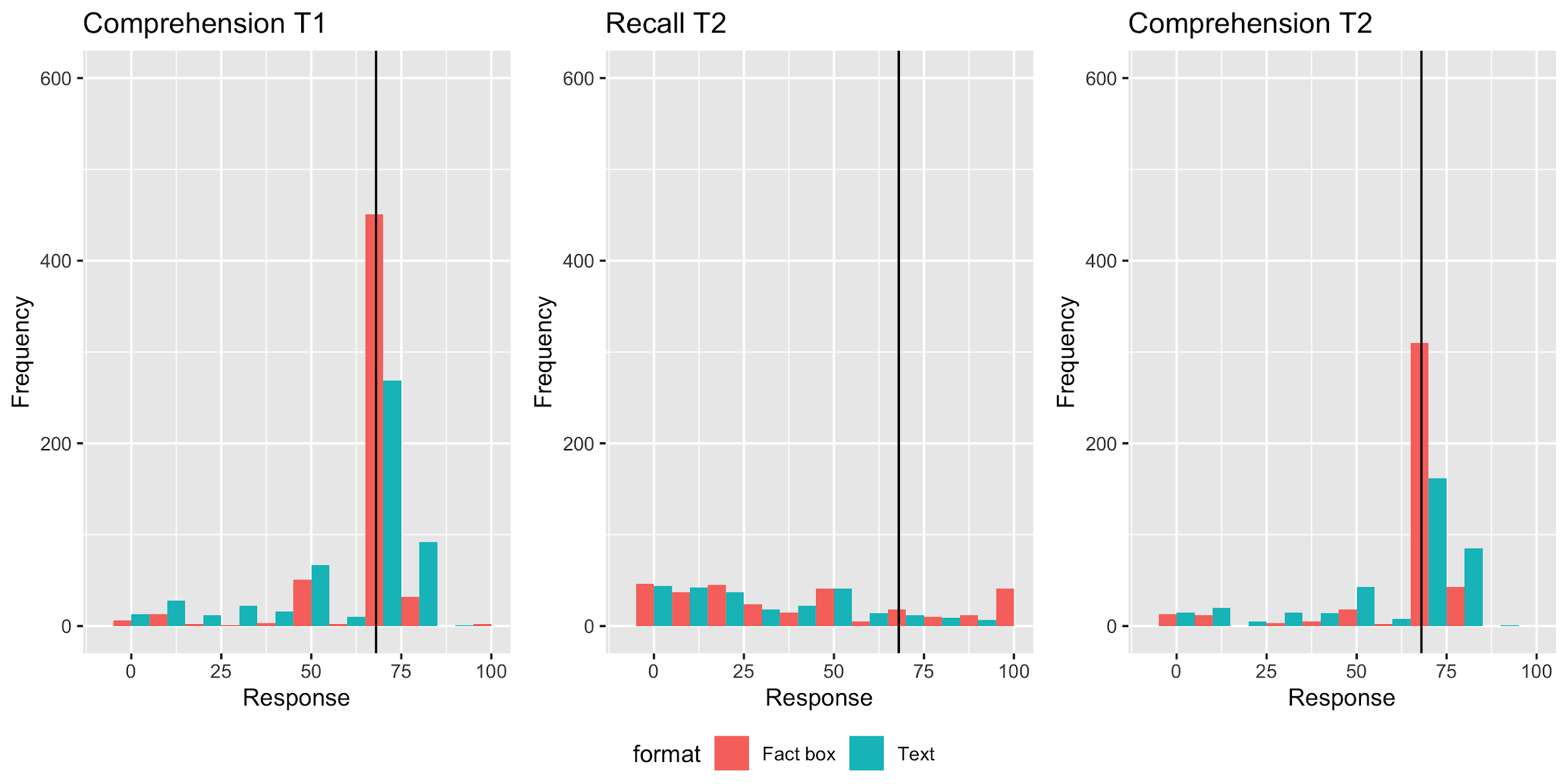
**

*Figure S7*. Histograms of open-response comprehension items by time point and format. Correct answers are shown with vertical black lines. Some outliers are not shown outside the x-axis range.
