# Supplementary material for: Risk communication in tables versus text: a registered report randomized trial on ‘fact boxes'
Source: R Soc Open Sci. 2020 Mar 25;7(3):190876. doi: 10.1098/rsos.190876 (PMC7137953; doi:10.1098/rsos.190876)
Supplement: Questionnaire - Baseline [file rsos190876supp2.docx]

Cambridge

{page info}

Before you decide whether to take part in this study it is important for you to understand why the research is being done and what it will involve. Please take time to read the following information carefully and discuss it with others if you wish. A member of the research team can be contacted if there is anything that is not clear or if you would like more information. Take time to decide whether or not you wish to take part.

**What is the purpose of the study?**

This study is designed to investigate how people make judgements about risk and evidence.

**Why have I been approached about this study?**

You have been invited to take part because we are looking for a broad range of participants.

**Do I have to take part?**

No, your participation is entirely voluntary. If you do decide to take part, you can withdraw at any time and without giving a reason.

**What will happen if I agree to take part?**

You will be asked to complete an online study designed to help us understand how people make judgments and decisions. You will also be asked to provide some general information about yourself (such as age) and to complete some questionnaires and to complete some problem-solving or memory tasks. The study will take about 15 minutes to complete. Detailed instructions will be given to you before you start the task. In two weeks, you will complete a similar study as a follow-up to test for changes over time.

**What are the advantages and disadvantages of taking part?**

There are no direct benefits to you from taking part. We hope that the data you provide will be useful in helping us understand more about human decision-making. There are no known risks of participation.

**Will my taking part in this project be kept confidential?**

We will not collect any personally identifiable information. For information about how the University uses personal data, please see: https://www.information-compliance.admin.cam.ac.uk/data-protection/research-participant-data.

**Data protection**

We will not collect any personally identifiable information. Any data you provide during the research (e.g., surveys) will only have anonymous data. The anonymous data will be downloaded and stored on servers that may be outside of the European Union (e.g., in a password-protected Dropbox account). The anonymous data may be presented at scientific conferences, published in scientific journals or shared with other researchers at academic institutions. You can withdraw your consent by indicating that in the study, and your data will be discarded.

**What happens to the results?**

The results of this study may help us learn how best to communicate information about benefits, risks, and evidence in ways that are clear and easy to understand. The results may also be presented at conferences and written up in scientific journals so that other researchers can build on this work. Any data we publish will be anonymised.

**Who is organising and funding the research?**

This study is funded by the Winton Centre for Risk and Evidence Communication at the University of Cambridge.

**Ethical review of the study**

The project has received ethical approval from the Psychology Research Ethics Committee of the University of Cambridge.

**Contact for further information**

If you have any questions you can contact Dr Cameron Brick, Department of Psychology, cb954@cam.ac.uk, telephone: 01223 764256 or William Skylark, Department of Psychology, wjm22@cam.ac.uk, telephone: 01223 333572.

**More about how we use your research data**

Cambridge University is the sponsor for this study based in the United Kingdom. We will be using information from you in order to undertake this study and will act as the data controller for this. Cambridge University will keep any identifiable information about you for 10 years after the study has finished. Your rights to access, change or move your information are limited, as we need to manage your information in specific ways in order for the research to be reliable and accurate. If you withdraw from the study, we will keep the information about you that we have already obtained, but exclude it from analyses and scientific manuscripts and presentations. To safeguard your rights, we will use the minimum personally identifiable information possible.

You can find out more about how we use your information go to https://www.medschl.cam.ac.uk/research/privacy-notice-how-we-use-your-research-data/ Health and care research should serve the public interest, which means that we have to demonstrate that our research serves the interests of society as a whole. We do this by following the UK Policy Framework for Health and Social Care Research. For general information about how we use your data: https://www.information-compliance.admin.cam.ac.uk/data-protection/research-participant-data

{end page info}

{page consent}

I have read and understand the Participant Information Sheet. I have had the opportunity to ask questions and had them answered. I understand that my participation is voluntary and that I am free to withdraw at any time without giving a reason. I understand that the data I provide may be stored and used for future research.

If you have any questions you can contact Dr Cameron Brick, Department of Psychology, cb954@cam.ac.uk, telephone: 01223 764256.

[UCconsent] Please tick the box below to indicate whether you agree or disagree to participate and have your data used as outlined on the previous pages.

<1> I agree to participate

<2> I do not agree to participate

{end page consent}

{module survey if UCconsent==1}

{page intro}

In this study you will be shown information collected by the University of Cambridge about a medical issue and asked to imagine that you are dealing with this problem and looking for information. After reading the message, you will be asked multiple-choice questions to see how well you understand the information. Please do not rush, because we use the results of these studies to design actual health messages used by patients. Our research group at Cambridge University produces tools used by tens of thousands of patients each month. Thank you for your help.<br><br> First, please indicate your feelings towards the following health procedures.

{end page intro}

{module att_mod order=randomize}

[att_vac] {grid roworder=randomize} What do you think about the influenza (flu) vaccine?

-[att_vac_effective] Effective

-[att_vac_safe] Safe

-[att_vac_science] Based on high-quality science

<1> Not at all

<2> A little

<3> A moderate amount

<4> A lot

<5> Very much

[att_teeth] {grid roworder=randomize} What do you think about teeth cleaning?

-[att_teeth_effective] Effective

-[att_teeth_safe] Safe

-[att_teeth_science] Based on high-quality science

<1> Not at all

<2> A little

<3> A moderate amount

<4> A lot

<5> Very much

[att_gene] {grid roworder=randomize} What do you think about genetic testing?

-[att_gene_effective] Effective

-[att_gene_safe] Safe

-[att_gene_science] Based on high-quality science

<1> Not at all

<2> A little

<3> A moderate amount

<4> A lot

<5> Very much

[att_antib] {grid roworder=randomize} What do you think about antibiotics?

-[att_antib_effective] Effective

-[att_antib_safe] Safe

-[att_antib_science] Based on high-quality science

<1> Not at all

<2> A little

<3> A moderate amount

<4> A lot

<5> Very much

{end module att_mod}

{page split}

{

Cambridgesplit = random(1,4)

}

{end page split}

{module ear1 if Cambridgesplit==1}


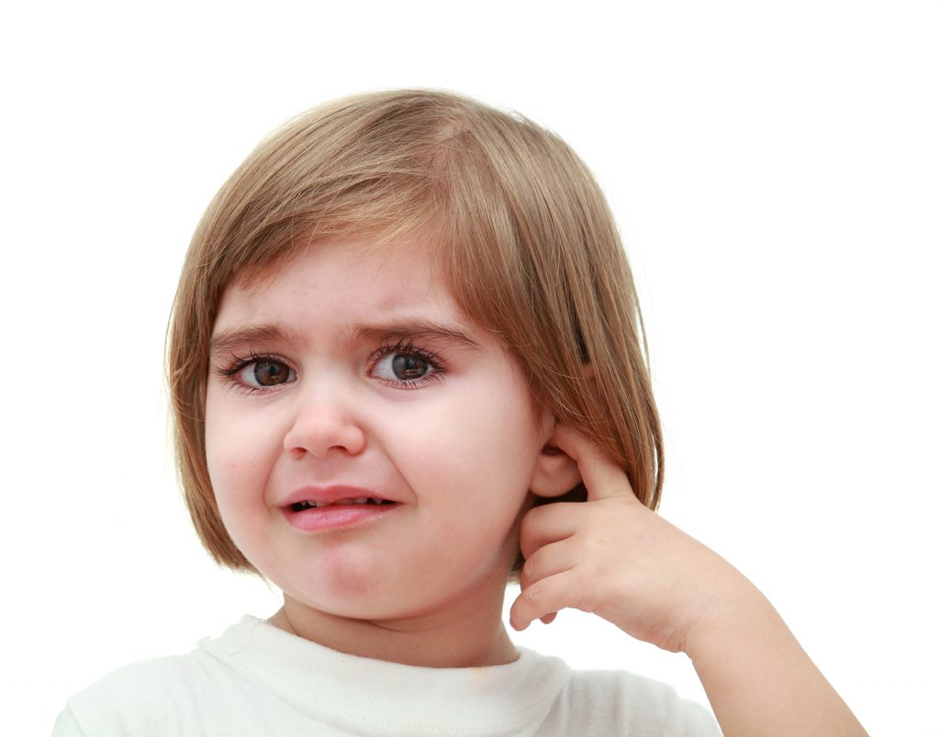


</br>

Imagine that a child you know is experiencing frequent ear infections.

Ear infections are a common medical problem, especially in children, and can cause discomfort and hearing loss. Would you recommend the child receives antibiotics or not?<br><br> Imagine that you look up information and see studies about whether antibiotics make ear infections clear up faster, or be less severe. The studies also looked at how many children experienced harms from the antibiotics such as vomiting, diarrhoea, or rash.<br><br> The studies compared how much better or worse off children were taking antibiotics compared with taking a placebo (a sugar pill: no medicine).<br><br> Next, you will see a summary of the research. Assume you found the following information about antibiotics for ear infections in children. Please read the information to consider whether or not you would decide to choose antibiotic treatment for the child.

{page earfactbox}

Assume you found the following information about antibiotics for treating inner ear infections in children. Please read the information to consider whether or not you would decide to choose antibiotic treatment for your child.<br><br>

The numbers below are for children 0-15 years of age with an acute middle ear infection who either received antibiotics or placebo (sugar pill) over a period of 7-14 days. <br><br>

Based on this information, please answer some questions. Do not rush: we will use the results of these studies to design messages for actual patients. Thank you for your help.<br><br>


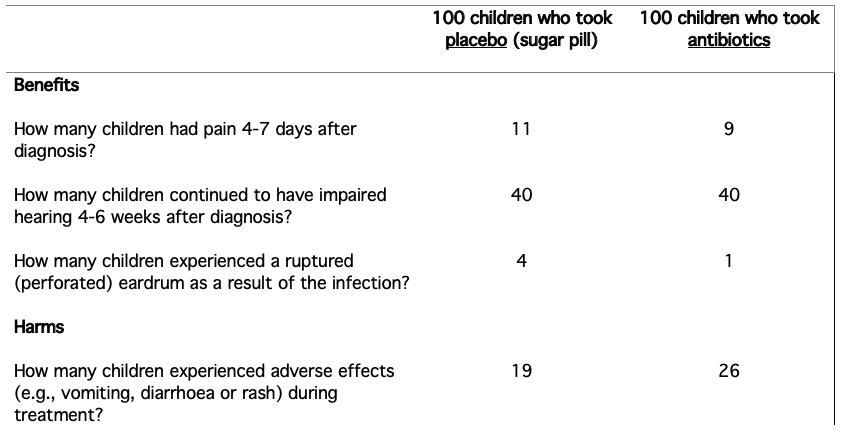


</br>

On the following pages, you will be shown this again and will answer questions about the content.

</br>

{end page earfactbox}

{page ear1x}


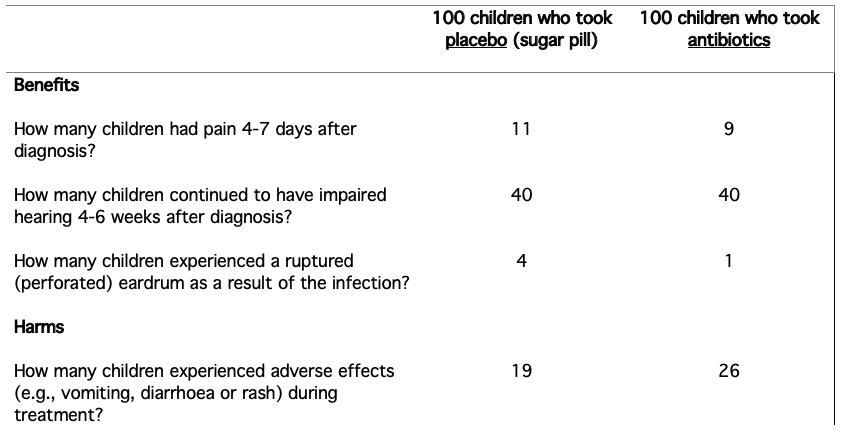
</br>

[c_ear_f1x] {open-int min=0 max=100} Out of 100 children with a middle ear infection who took antibiotics, how many experienced a ruptured eardrum?

[c_ear_f9x] {open-int min=0 max=100} Out of 100 children with a middle ear infection who took antibiotics, how many experienced an adverse effect (e.g., vomiting, diarrhoea or rash)?

[c_ear_f10x] {open-int min=0 max=100} Out of 100 children with a middle ear infection who took placebo, how many continued to have impaired hearing 4-6 weeks after diagnosis?

[c_ear_f2] Children who took which treatment had a higher chance of developing a ruptured eardrum?

<1> Antibiotics

<2> Both antibiotics and placebo were the same

<3> Placebo

<4> This information is not shown

<5> I don't know

{end page ear1x}

{page ear2x}


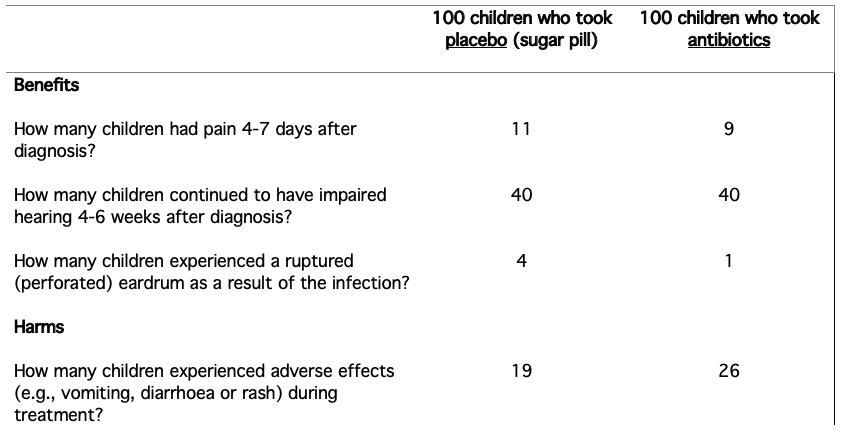
</br>

[c_ear_f3] Children who took which treatment had a higher chance of experiencing pain 4-7 days after diagnosis?

<1> Antibiotics

<2> Both antibiotics and placebo were the same

<3> Placebo

<4> This information is not shown

<5> I don't know

[c_ear_f4] Out of 100 children who received placebo, how many had issues with hearing 4-6 weeks after diagnosis?

<1> Almost none

<2> About a quarter

<3> About half

<4> About three quarters

<5> I don't know

[c_ear_f12] Which result was less common in children who took antibiotics compared with those who took a placebo?

<1> Pain

<2> Hearing issues

<3> Adverse effects

<4> This information is not shown

<5> I don't know

[c_ear_f5] How did antibiotics affect children's hearing 4-6 weeks after diagnosis compared to placebo?

<1> Antibiotics REDUCED hearing

<2> Antibiotics IMPROVED hearing

<3> The effect was the same in both groups

<4> This information is not shown

<5> I don't know

{end page ear2x}

{page ear3x}


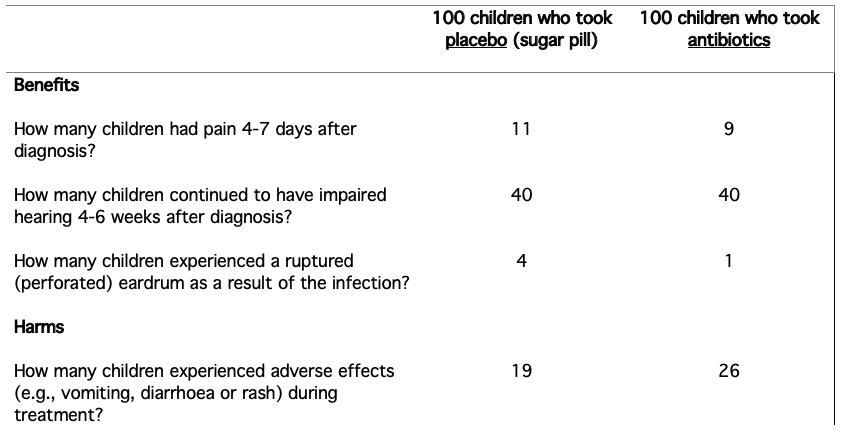


[c_ear_f6] Which of these statements best describes the evidence shown here?

<1> Antiobiotics had no effect

<2> Antibiotics only caused harm

<3> Antibiotic only caused benefits

<4> Antibiotics caused both harm and benefits

<5> I don't know

[c_ear_f11] Which group was less likely to experience pain 4-7 days after diagnosis?

<1> Children who took antibiotics

<2> Children who took a placebo

<3> The effect was the same in both groups

<4> This information was not shown

<5> I don't know

[c_ear_f7] Which group experienced more adverse effects (harms) such as vomiting, diarrhoea or rash?

<1> Children who took antibiotics

<2> Children who took a placebo

<3> The effect was the same in both groups

<4> This information was not shown

<5> I don't know

[c_ear_f8x] {open-int min=0 max=100} How many more of the 100 children taking antibiotics experienced adverse effects (harms) compared to the 100 children taking placebo? Enter 0 if there was no difference.

{end page ear3x}

[decision_ear] Imagine that a child you know is experiencing frequent ear infections. Based on the information you saw, please consider what decision you personally make about them taking antibiotics.

<1> The child should take antibiotics

<2> The child should NOT take antibiotics

<3> It would not matter if they take antibiotics or not because the results are similar

<4> Unsure

[conflict_ear] {grid roworder=randomize} Now, thinking about the choice you just made and the information you read, please look at the following comments some people make when deciding about treatment. Please show how strongly you agree or disagree with these comments.

-[conflict_ear1] I know which options are available to me

-[conflict_ear2] I know the benefits of each option

-[conflict_ear3] I know the risks and side effects of each option

<1> Strongly disagree

<2> Disagree

<3> Somewhat disagree

<4> Neither agree nor disagree

<5> Somewhat agree

<6> Agree

<7> Strongly agree

{end module ear1}

{module ear2 if Cambridgesplit==2}


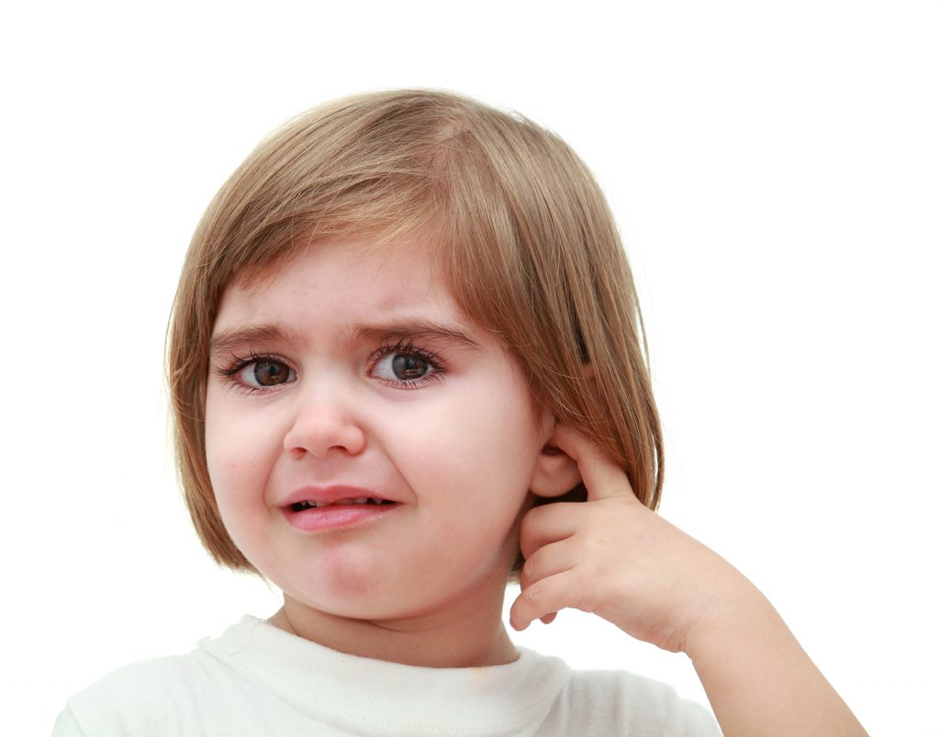


</br>

Imagine that a child you know is experiencing frequent ear infections.

Ear infections are a common medical problem, especially in children, and can cause discomfort and hearing loss. Would you recommend the child receives antibiotics or not?<br><br> Imagine that you look up information and see studies about whether antibiotics make ear infections clear up faster, or be less severe. The studies also looked at how many children experienced harms from the antibiotics such as vomiting, diarrhoea, or rash.<br><br> The studies compared how much better or worse off children were taking antibiotics compared with taking a placebo (a sugar pill: no medicine).<br><br> Next, you will see a summary of the research. Assume you found the following information about antibiotics for ear infections in children. Please read the information to consider whether or not you would decide to choose antibiotic treatment for the child.

{page earfactboxtext2}

Assume you found the following information about antibiotics for treating inner ear infections in children. Please read the information to consider whether or not you would decide to choose antibiotic treatment for your child.<br><br>

The numbers below are for children 0-15 years of age with an acute middle ear infection who either received antibiotics or placebo (sugar pill) over a period of 7-14 days. <br><br>

Based on this information, please answer some questions. Do not rush: we will use the results of these studies to design messages for actual patients. Thank you for your help.<br><br>

Benefits: Of the 100 children who took antibiotics, 9 children had pain 4-7 days after diagnosis compared to 11 out of the 100 children who took the placebo (sugar pill). There was no difference between groups in how many children had impaired hearing 4-6 weeks after diagnosis (40 in each group). One out of 100 children who took antibiotics had a ruptured (perforated) eardrum as a result of the infection, compared to 4 out of 100 children who took placebo.<br><br>

Harms: 26 out of 100 children taking antibiotics experienced an adverse effect (e.g., vomiting, diarrhoea or rash), compared to 19 out of 100 children who took the placebo.<br><br>

On the following pages, you will be shown this again and will answer questions about the content.

</br>

{end page earfactboxtext2}

{page ear4x}

Benefits: Of the 100 children who took antibiotics, 9 children had pain 4-7 days after diagnosis compared to 11 out of the 100 children who took the placebo (sugar pill). There was no difference between groups in how many children had impaired hearing 4-6 weeks after diagnosis (40 in each group). One out of 100 children who took antibiotics had a ruptured (perforated) eardrum as a result of the infection, compared to 4 out of 100 children who took placebo.<br><br>

Harms: 26 out of 100 children taking antibiotics experienced an adverse effect (e.g., vomiting, diarrhoea or rash), compared to 19 out of 100 children who took the placebo.<br><br>

[c_ear_t1x] {open-int min=0 max=100} Out of 100 children with a middle ear infection who took antibiotics, how many experienced a ruptured eardrum?

[c_ear_t9x] {open-int min=0 max=100} Out of 100 children with a middle ear infection who took antibiotics, how many experienced an adverse effect (e.g., vomiting, diarrhoea or rash)?

[c_ear_t10x] {open-int min=0 max=100} Out of 100 children with a middle ear infection who took placebo, how many continued to have impaired hearing 4-6 weeks after diagnosis?

[c_ear_t2] Children who took which treatment had a higher chance of developing a ruptured eardrum?

<1> Antibiotics

<2> Both antibiotics and placebo were the same

<3> Placebo

<4> This information is not shown

<5> I don't know

{end page ear4x}

{page ear5x}

Benefits: Of the 100 children who took antibiotics, 9 children had pain 4-7 days after diagnosis compared to 11 out of the 100 children who took the placebo (sugar pill). There was no difference between groups in how many children had impaired hearing 4-6 weeks after diagnosis (40 in each group). One out of 100 children who took antibiotics had a ruptured (perforated) eardrum as a result of the infection, compared to 4 out of 100 children who took placebo.<br><br>

Harms: 26 out of 100 children taking antibiotics experienced an adverse effect (e.g., vomiting, diarrhoea or rash), compared to 19 out of 100 children who took the placebo.<br><br>

[c_ear_t3] Children who took which treatment had a higher chance of experiencing pain 4-7 days after diagnosis?

<1> Antibiotics

<2> Both antibiotics and placebo were the same

<3> Placebo

<4> This information is not shown

<5> I don't know

[c_ear_t4] Out of 100 children who received placebo, how many had issues with hearing 4-6 weeks after diagnosis?

<1> Almost none

<2> About a quarter

<3> About half

<4> About three quarters

<5> I don't know

[c_ear_t12] Which result was less common in children who took antibiotics compared with those who took a placebo?

<1> Pain

<2> Hearing issues

<3> Adverse effects

<4> This information is not shown

<5> I don't know

[c_ear_t5] How did antibiotics affect children's hearing 4-6 weeks after diagnosis compared to placebo?

<1> Antibiotics REDUCED hearing

<2> Antibiotics IMPROVED hearing

<3> The effect was the same in both groups

<4> This information is not shown

<5> I don't know

{end page ear5x}

{page ear6x}

Benefits: Of the 100 children who took antibiotics, 9 children had pain 4-7 days after diagnosis compared to 11 out of the 100 children who took the placebo (sugar pill). There was no difference between groups in how many children had impaired hearing 4-6 weeks after diagnosis (40 in each group). One out of 100 children who took antibiotics had a ruptured (perforated) eardrum as a result of the infection, compared to 4 out of 100 children who took placebo.<br><br>

Harms: 26 out of 100 children taking antibiotics experienced an adverse effect (e.g., vomiting, diarrhoea or rash), compared to 19 out of 100 children who took the placebo.<br><br>

[c_ear_t6] Which of these statements best describes the evidence shown here?

<1> Antiobiotics had no effect

<2> Antibiotics only caused harm

<3> Antibiotic only caused benefits

<4> Antibiotics caused both harm and benefits

<5> I don't know

[c_ear_t11] Which group was less likely to experience pain 4-7 days after diagnosis?

<1> Children who took antibiotics

<2> Children who took a placebo

<3> The effect was the same in both groups

<4> This information was not shown

<5> I don't know

[c_ear_t7] Which group experienced more adverse effects (harms) such as vomiting, diarrhoea or rash?

<1> Children who took antibiotics

<2> Children who took a placebo

<3> The effect was the same in both groups

<4> This information was not shown

<5> I don't know

[c_ear_t8x] {open-int min=0 max=100} How many more of the 100 children taking antibiotics experienced adverse effects (harms) compared to the 100 children taking placebo? Enter 0 if there was no difference.

{end page ear6x}

[decision_eart] Imagine that a child you know is experiencing frequent ear infections. Based on the information you saw, please consider what decision you personally make about them taking antibiotics.

<1> The child should take antibiotics

<2> The child should NOT take antibiotics

<3> It would not matter if they take antibiotics or not because the results are similar

<4> Unsure

[conflict_eart] {grid roworder=randomize} Now, thinking about the choice you just made and the information you read, please look at the following comments some people make when deciding about treatment. Please show how strongly you agree or disagree with these comments.

-[conflict_eart1] I know which options are available to me

-[conflict_eart2] I know the benefits of each option

-[conflict_eart3] I know the risks and side effects of each option

<1> Strongly disagree

<2> Disagree

<3> Somewhat disagree

<4> Neither agree nor disagree

<5> Somewhat agree

<6> Agree

<7> Strongly agree

{end module ear2}

{module flu1 if Cambridgesplit==3}


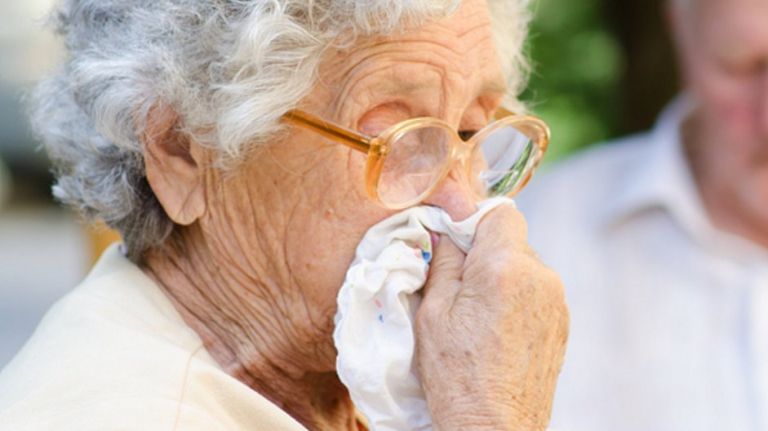


</br>

Imagine that an older relative of yours has been offered an influenza vaccination.<br><br> Influenza is a viral illness that is particularly dangerous in vulnerable populations such as children and the elderly, and can develop into serious illness. Would you recommend your older relative is vaccinated or not? <br><br> Imagine that you look up information and see studies about whether the influenza vaccine reduces the likelihood and severity of flu symptoms in people over 60. The studies also compared how many of these people experienced harms from the vaccine such as pain, tenderness, or swelling at the injection site.<br><br> The studies compared how much better or worse off older adults were who took the influenza vaccine compared to those who took placebo (a dummy injection—no vaccine).<br><br> The variation in the effectiveness of the influenza vaccination depends on where and how severely the virus spreads and on how well the seasonal vaccine matches the circulating strains. Because the virus varies each year, an annual vaccination is necessary.<br><br> Next, you will see a summary of the research. Assume you found the following information about influenza vaccines for older adults. Please read the information to consider whether or not you would decide to recommend a vaccine for an older relative.

{page flufactbox}

Assume you found the following information about vaccines to prevent influenza in older adults. Please read the information to consider whether or not you would decide to recommend a vaccine for an older relative.<br><br>

The numbers below are for adults aged 60 or older who were observed for one year. Older adults with placebo received an injection with a saline solution (no vaccine) instead of the influenza vaccine.

Based on this information, please answer some questions. Do not rush: we will use the results of these studies to design messages for actual patients. Thank you for your help.<br><br>


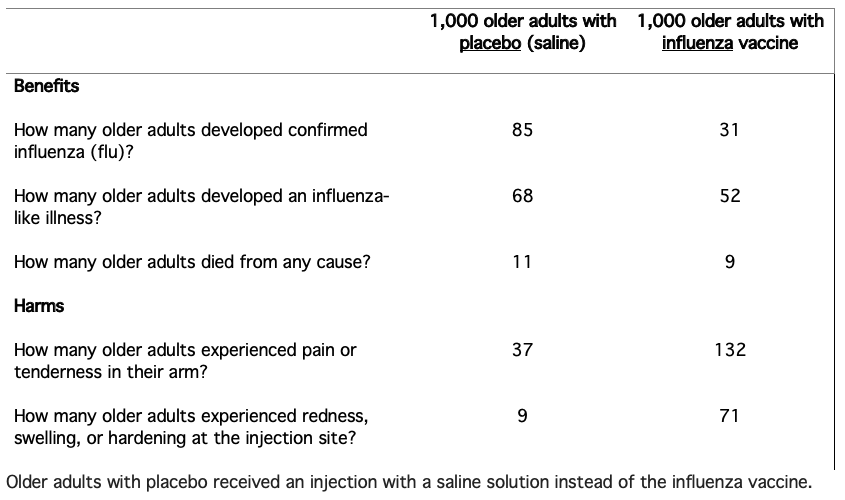


</br>

On the following pages, you will be shown this again and will answer questions about the content.

{end page flufactbox}

{page flu1x}


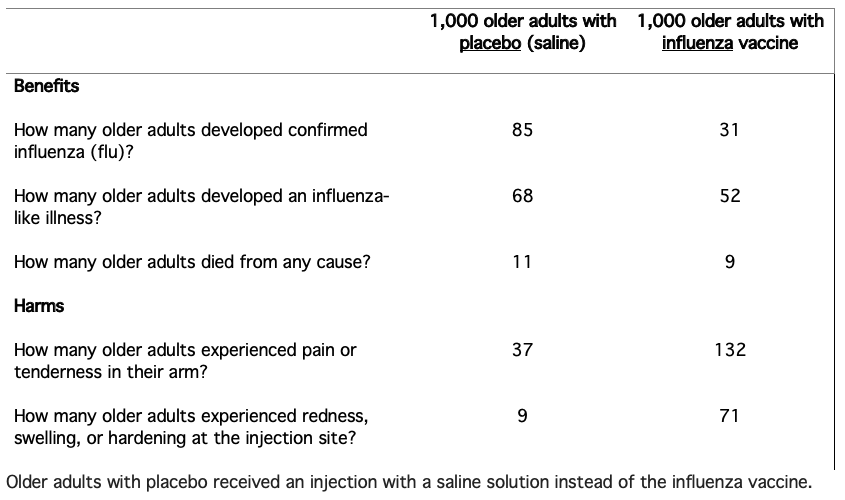


</br>

[c_flu_f1x] {open-int min=0 max=1000} Out of 1,000 adults aged 60 or older who received the influenza vaccine, how many died from any cause within one year?

[c_flu_f9x] {open-int min=0 max=1000} Out of 1,000 adults aged 60 or older who received the influenza vaccine, how many experienced pain or tenderness in their arm?

[c_flu_f10x] {open-int min=0 max=1000} Out of 1,000 adults aged 60 or older who received the placebo, how many developed an influenza-like illness?

[c_flu_f2] Older adults who took which treatment had a higher chance of dying within one year?

<1> Influenza vaccine

<2> Both influenza vaccine and placebo were the same

<3> Placebo

<4> This information is not shown

<5> I don't know

{end page flu1x}

{page flu2x}


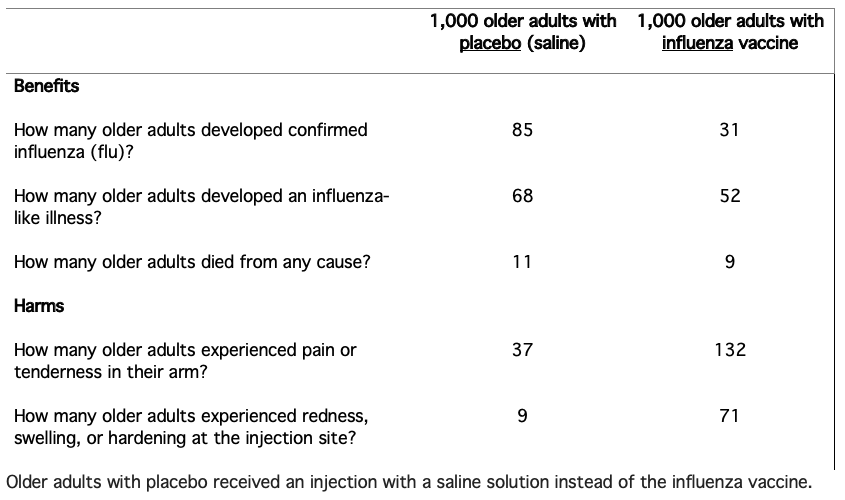


</br>

[c_flu_f3] Older adults who took which treatment had a higher chance of developing influenza in the following year?

<1> Influenza vaccine

<2> Both influenza vaccine and placebo were the same

<3> Placebo

<4> This information is not shown

<5> I don't know

[c_flu_f4x] {open-int min=0 max=1000} Out of 1,000 adults aged 60 or older who received placebo, how many experienced redness, swelling, or hardening at the injection site?

[c_flu_f12] Which result was more common in adults who received the influenza vaccine compared with those who took a placebo?

<1> Confirmed influenza

<2> Dying of any cause

<3> Pain or tenderness

<4> This information is not shown

<5> I don't know

[c_flu_f5] How did receiving the influenza vaccine affect how many older adults developed an influenza-like illness compared with placebo?

<1> The influenza vaccine REDUCED influenza-like illnesses

<2> The influenza vaccine INCREASED influenza-like illnesses

<3> The effect was the same in both groups

<4> This information is not shown

<5> I don't know

{end page flu2x}

{page flu3x}


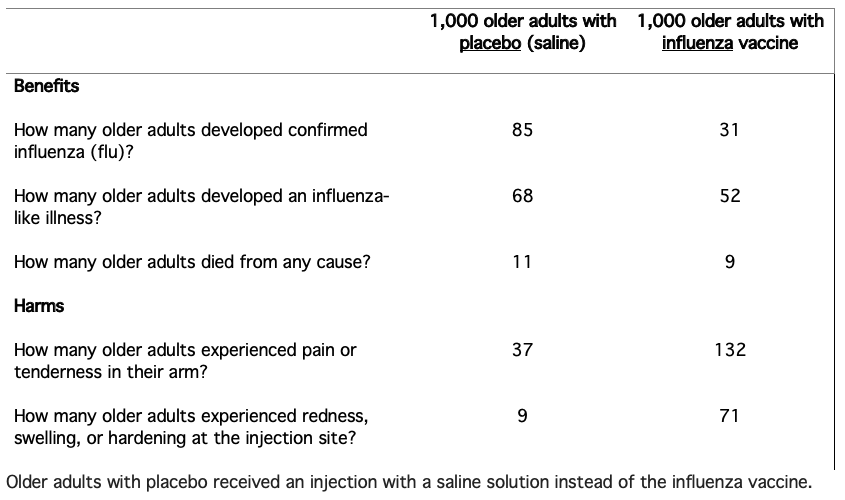


</br>

[c_flu_f6] Which of these statements best describes the evidence shown here?

<1> The influenza vaccine had no effect

<2> The influenza vaccine only caused harm

<3> The influenza vaccine only caused benefits

<4> The influenza vaccine caused both harm and benefits

<5> I don't know

[c_flu_f11] Which group was less likely to develop confirmed influenza?

<1> Older adults who took the influenza vaccine

<2> Older adults who took a placebo

<3> The effect was the same in both groups

<4> This information was not shown

<5> I don't know

[c_flu_f7] Which group experienced more harms (such as pain or tenderness in the arm)?

<1> Older adults who took the influenza vaccine

<2> Older adults who took a placebo

<3> The effect was the same in both groups

<4> This information was not shown

<5> I don't know

[c_flu_f8] {open} How many more of the 1,000 older adults who received the influenza vaccine experienced redness, swelling, or hardening at the injection site compared to the 1,000 older adults who received a placebo?

{end page flu3x}

[decision_flu] Imagine that an older relative has been offered an influenza vaccination. Based on the information you saw, please consider what decision you personally would make about them getting the vaccine.

<1> The older adult should get the influenza vaccine

<2> The older adult should NOT get the influenza vaccine

<3> It would not matter if they get the vaccine or not because the results are similar

<4> Unsure

[conflict_flu] {grid roworder=randomize} Now, thinking about the choice you just made and the information you read, please look at the following comments some people make when deciding about treatment. Please show how strongly you agree or disagree with these comments.

-[conflict_flu1] I know which options are available to me

-[conflict_flu2] I know the benefits of each option

-[conflict_flu3] I know the risks and side effects of each option

<1> Strongly disagree

<2> Disagree

<3> Somewhat disagree

<4> Neither agree nor disagree

<5> Somewhat agree

<6> Agree

<7> Strongly agree

{end moduleflu1}

{module flu2 if Cambridgesplit==4}


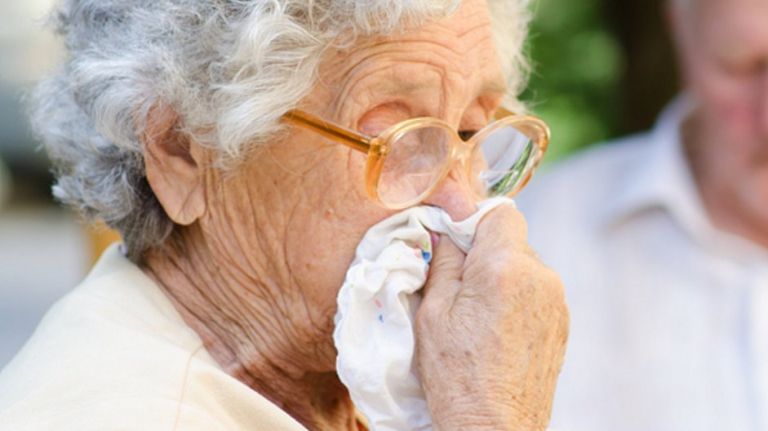


</br>

Imagine that an older relative of yours has been offered an influenza vaccination.<br><br> Influenza is a viral illness that is particularly dangerous in vulnerable populations such as children and the elderly, and can develop into serious illness. Would you recommend your older relative is vaccinated or not?<br><br> Imagine that you look up information and see studies about whether the influenza vaccine reduces the likelihood and severity of flu symptoms in people over 60. The studies also compared how many of these people experienced harms from the vaccine such as pain, tenderness, or swelling at the injection site.<br><br> The studies compared how much better or worse off older adults were who took the influenza vaccine compared to those who took placebo (a dummy injection—no vaccine).<br><br> The variation in the effectiveness of the influenza vaccination depends on where and how severely the virus spreads and on how well the seasonal vaccine matches the circulating strains. Because the virus varies each year, an annual vaccination is necessary.<br><br> Next, you will see a summary of the research. Assume you found the following information about influenza vaccines for older adults. Please read the information to consider whether or not you would decide to recommend a vaccine for an older relative.

{page flufactbox2}

Assume you found the following information about vaccines to prevent influenza in older adults. Please read the information to consider whether or not you would decide to recommend a vaccine for an older relative.<br><br>

The numbers below are for adults aged 60 or older who were observed for one year. Older adults with placebo received an injection with a saline solution (no vaccine) instead of the influenza vaccine.<br><br>

Based on this information, please answer some questions. Do not rush: we will use the results of these studies to design messages for actual patients. Thank you for your help.<br><br>

Benefits: Of 1,000 adults ages 60 and older who received the influenza vaccine, 31 developed confirmed influenza (flu) over the next year, compared to 85 out of 1,000 older adults who received a placebo (no vaccine). Of older adults receiving the influenza vaccine, 52 out of 1,000 developed an influenza-like illness, while 68 out of 1,000 did in the placebo group. In the influenza vaccine group, 9 out of 1,000 died of all causes over the next year, while 11 out of 1,000 died in the placebo group.<br><br> Harms: Of the 1,000 older adults receiving the influenza vaccine, 132 experienced pain or tenderness in their arm, compared to 37 out of 1,000 in the placebo group. In the influenza vaccine group, 71 out of 1,000 experienced redness, swelling, or hardening at the injection site, compared to 9 out of 1,000 in the placebo group.<br><br><br>

On the following pages, you will be shown this again and will answer questions about the content.

{end page flufactbox2}

{page flu4x}

Benefits: Of 1,000 adults ages 60 and older who received the influenza vaccine, 31 developed confirmed influenza (flu) over the next year, compared to 85 out of 1,000 older adults who received a placebo (no vaccine). Of older adults receiving the influenza vaccine, 52 out of 1,000 developed an influenza-like illness, while 68 out of 1,000 did in the placebo group. In the influenza vaccine group, 9 out of 1,000 died of all causes over the next year, while 11 out of 1,000 died in the placebo group.<br><br> Harms: Of the 1,000 older adults receiving the influenza vaccine, 132 experienced pain or tenderness in their arm, compared to 37 out of 1,000 in the placebo group. In the influenza vaccine group, 71 out of 1,000 experienced redness, swelling, or hardening at the injection site, compared to 9 out of 1,000 in the placebo group.<br><br><br>

[c_flu_t1x] {open-int min=0 max=1000} Out of 1,000 adults aged 60 or older who received the influenza vaccine, how many died from any cause within one year?

[c_flu_t9x] {open-int min=0 max=1000} Out of 1,000 adults aged 60 or older who received the influenza vaccine, how many experienced pain or tenderness in their arm?

[c_flu_t10x] {open-int min=0 max=1000} Out of 1,000 adults aged 60 or older who received the placebo, how many developed an influenza-like illness?

[c_flu_t2] Older adults who took which treatment had a higher chance of dying within one year?

<1> Influenza vaccine

<2> Both influenza vaccine and placebo were the same

<3> Placebo

<4> This information is not shown

<5> I don't know

{end page flu4x}

{page flu5x}

Benefits: Of 1,000 adults ages 60 and older who received the influenza vaccine, 31 developed confirmed influenza (flu) over the next year, compared to 85 out of 1,000 older adults who received a placebo (no vaccine). Of older adults receiving the influenza vaccine, 52 out of 1,000 developed an influenza-like illness, while 68 out of 1,000 did in the placebo group. In the influenza vaccine group, 9 out of 1,000 died of all causes over the next year, while 11 out of 1,000 died in the placebo group.<br><br> Harms: Of the 1,000 older adults receiving the influenza vaccine, 132 experienced pain or tenderness in their arm, compared to 37 out of 1,000 in the placebo group. In the influenza vaccine group, 71 out of 1,000 experienced redness, swelling, or hardening at the injection site, compared to 9 out of 1,000 in the placebo group.<br><br><br>

[c_flu_t3] Older adults who took which treatment had a higher chance of developing influenza in the following year?

<1> Influenza vaccine

<2> Both influenza vaccine and placebo were the same

<3> Placebo

<4> This information is not shown

<5> I don't know

[c_flu_t4x] {open-int min=0 max=1000} Out of 1,000 adults aged 60 or older who received placebo, how many experienced redness, swelling, or hardening at the injection site?

[c_flu_t12] Which result was more common in adults who received the influenza vaccine compared with those who took a placebo?

<1> Confirmed influenza

<2> Dying of any cause

<3> Pain or tenderness

<4> This information is not shown

<5> I don't know

[c_flu_t5] How did receiving the influenza vaccine affect how many older adults developed an influenza-like illness compared with placebo?

<1> The influenza vaccine REDUCED influenza-like illnesses

<2> The influenza vaccine INCREASED influenza-like illnesses

<3> The effect was the same in both groups

<4> This information is not shown

<5> I don't know

{end page flu5x}

{page flu6x}

Benefits: Of 1,000 adults ages 60 and older who received the influenza vaccine, 31 developed confirmed influenza (flu) over the next year, compared to 85 out of 1,000 older adults who received a placebo (no vaccine). Of older adults receiving the influenza vaccine, 52 out of 1,000 developed an influenza-like illness, while 68 out of 1,000 did in the placebo group. In the influenza vaccine group, 9 out of 1,000 died of all causes over the next year, while 11 out of 1,000 died in the placebo group.<br><br> Harms: Of the 1,000 older adults receiving the influenza vaccine, 132 experienced pain or tenderness in their arm, compared to 37 out of 1,000 in the placebo group. In the influenza vaccine group, 71 out of 1,000 experienced redness, swelling, or hardening at the injection site, compared to 9 out of 1,000 in the placebo group.<br><br><br>

[c_flu_t6] Which of these statements best describes the evidence shown here?

<1> The influenza vaccine had no effect

<2> The influenza vaccine only caused harm

<3> The influenza vaccine only caused benefits

<4> The influenza vaccine caused both harm and benefits

<5> I don't know

[c_flu_t11] Which group was less likely to develop confirmed influenza?

<1> Older adults who took the influenza vaccine

<2> Older adults who took a placebo

<3> The effect was the same in both groups

<4> This information was not shown

<5> I don't know

[c_flu_t7] Which group experienced more harms (such as pain or tenderness in the arm)?

<1> Older adults who took the influenza vaccine

<2> Older adults who took a placebo

<3> The effect was the same in both groups

<4> This information was not shown

<5> I don't know

[c_flu_t8x] {open-int min=0 max=1000} How many more of the 1,000 older adults who received the influenza vaccine experienced redness, swelling, or hardening at the injection site compared to the 1,000 older adults who received a placebo?

{end page flu6x}

[decision_flut] Imagine that an older relative has been offered an influenza vaccination. Based on the information you saw, please consider what decision you personally would make about them getting the vaccine.

<1> The older adult should get the influenza vaccine

<2> The older adult should NOT get the influenza vaccine

<3> It would not matter if they get the vaccine or not because the results are similar

<4> Unsure

[conflict_flut] {grid roworder=randomize} Now, thinking about the choice you just made and the information you read, please look at the following comments some people make when deciding about treatment. Please show how strongly you agree or disagree with these comments.

-[conflict_flu1t] I know which options are available to me

-[conflict_flu2t] I know the benefits of each option

-[conflict_flu3t] I know the risks and side effects of each option

<1> Strongly disagree

<2> Disagree

<3> Somewhat disagree

<4> Neither agree nor disagree

<5> Somewhat agree

<6> Agree

<7> Strongly agree

{end moduleflu2}

[engagement] {grid roworder=randomize} Next, recall the information you were shown about harms and benefits.

-[engagement1] Would other people want to read this?

-[engagement2] Are you interested in this information?

-[engagement3] Do you like how this information is presented?

<1> Not at all

<2> A little

<3> Somewhat

<4> A fair amount

<5> Very much

[trust1] How reliable do you think these numbers are?

<1> 1 - Not at all

<2> 2

<3> 3

<4> 4

<5> 5 - Very reliable

[trust2] How trustworthy do you think these numbers are?

<1> 1 - Not at all

<2> 2

<3> 3

<4> 4

<5> 5 - Very trustworthy

[comments] {open rows=15 cols=60 required=NONE} Do you have any other comments about the information you were shown?

{page puzzle}

Please answer the puzzle questions below. Do not use a calculator but feel free to use scrap paper to make notes.

{end page puzzle}

[num1a] {open-int min=0 max=100} Out of 1,000 people in a small town 500 are members of a choir. Out of these 500 members in the choir 100 are men. Out of the 500 inhabitants that are not in the choir 300 are men. What is the probability that a randomly drawn man is a member of the choir? Please indicate the probability in percent.

[num2a if num1a!=25] {open-int min=0 max=50} Imagine we are throwing a five-sided die 50 times. On average, out of these 50 throws how many times would this five sided die show an odd number (1, 3, or 5)?

[num3a if num1a==25] {open-int min=0 max=70} Imagine we are throwing a loaded die (6 sides) 70 times. The probability that the die shows a 6 is twice as high as the probability of each of the other numbers. On average, out of these 70 throws how many times would the die show the number 6?

[num4a if num3a!=20] {open-int min=0 max=100} In a forest 20% of mushrooms are red, 50% brown and 30% white. A red mushroom is poisonous with a probability of 20%. A mushroom that is not red is poisonous with a probability of 5%. What is the probability that a poisonous mushroom in the forest is red?

{page ladder}


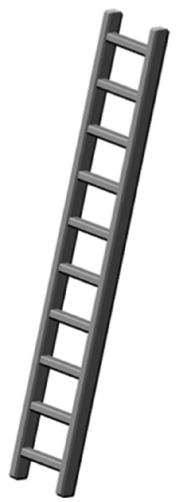
[status2]Think of a ladder as representing where people stand in their communities. People define community in different ways; please define it in whatever way is most meaningful to you. At the **top** of the ladder are the people who have the highest standing in the community. At the **bottom** are the people who have the lowest standing in the community.

Where would you place yourself on this ladder?**<br><br>Please select the number between 1 and 10, where 1 is the top rung of the ladder and 10 is the bottom rung of the ladder.

<1> 1

<2> 2

<3> 3

<4> 4

<5> 5

<6> 6

<7> 7

<8> 8

<9> 9

<10> 10

{end page ladder}

[health] Please rate your general health.

<1> Excellent

<2> Good

<3> Fair

<4> Poor

[comments2] {open rows=15 cols=60 required=NONE} Did you have any problems with the survey? Any technical issues or problems with questions? Other comments for our team?

{page debrief}

The first time point is complete. The study has two parts. We depend on participants completing the follow-up. PLEASE look for a follow-up email in one month and complete the next part. Thank you.

{end page debrief}

{end module survey}
