# Supplementary material for: Risk communication in tables versus text: a registered report randomized trial on ‘fact boxes'
Source: R Soc Open Sci. 2020 Mar 25;7(3):190876. doi: 10.1098/rsos.190876 (PMC7137953; doi:10.1098/rsos.190876)
Supplement: Questionnaire - Follow-up [file rsos190876supp3.docx]

Cambridge Wave 2

{page info}

Thank you for starting this study, which is a follow-up to the study that you completed one month ago.

{end page info}

{page introstart}

This survey is on a variety of topics, and the results will be used to inform our clients.

Your YouGov Account will be credited with 50 points for completing the survey.

We have tested the survey and found that, on average it takes around 14 to 16 minutes to complete. This time may vary depending on factors such as your Internet connection speed and the answers you give.

Please click the forward button below to continue.

{end page introstart}

{page split}

{

if history.P_Cambridge_healthinfo.Cambridgesplit: Cambridgesplit = history.P_Cambridge_healthinfo.Cambridgesplit

else: Cambridgesplit = random(1,4)

}

{end page split}

{module ear1 if Cambridgesplit==1}


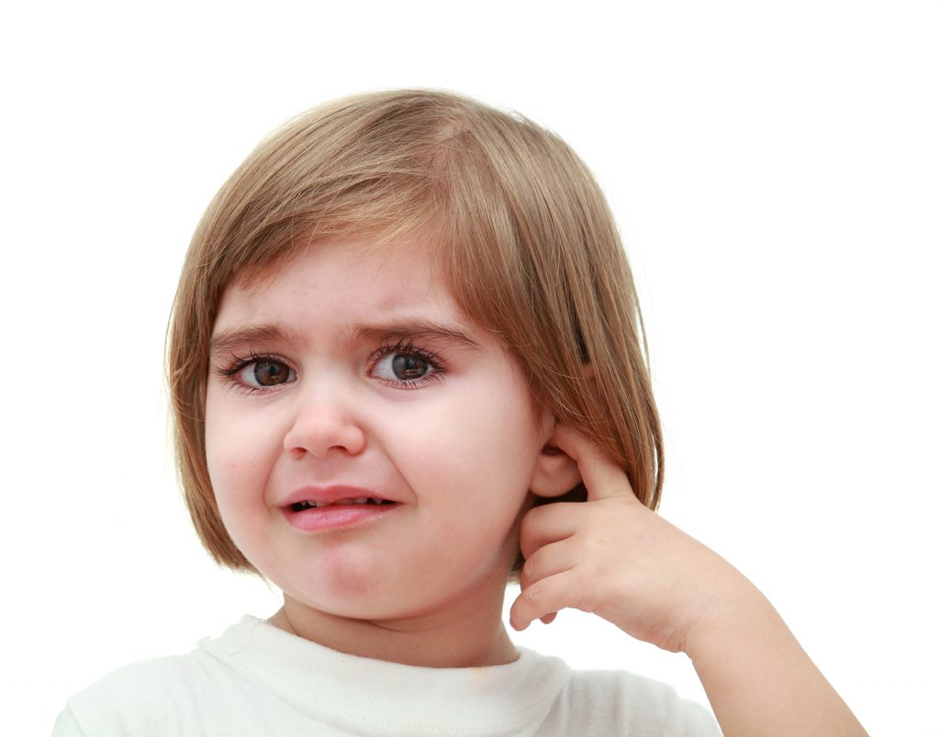


Ear infections are a common medical problem, especially in children, and can cause discomfort and hearing loss.<br><br>

[decision_ear] Imagine that a child you know is experiencing frequent ear infections. Based on what you remember, please consider what decision you would personally make about them taking antibiotics.

<1> The child should take antibiotics

<2> The child should NOT take antibiotics

<3> It would not matter if they take antibiotics or not because the results are similar

<4> Unsure

{page earfactbox_recall}

In the first part of the study a month ago, you saw information on antibiotics for children with ear infections. We provided you information on studies about whether antibiotics make ear infections clear up faster, or be less severe. The studies also looked at how many children experienced harms from the antibiotics such as vomiting, diarrhoea, or rash.<br><br>

On the following pages we would like to ask you some questions about what you remember. The information from last month showed outcomes for a group who took antibiotics compared to those who took a placebo (a sugar pill).<br><br>

Please enter a guess for each answer, even if you don't remember. Please try for accuracy, but we don't expect you to remember all the details.

{end page earfactbox_recall}

{page ear1x_recall}

[c_ear_f1_recall] {open-int min=0 max=100} Out of 100 children with a middle ear infection who took antibiotics, how many experienced a ruptured eardrum?

[c_ear_f9_recall] {open-int min=0 max=100} Out of 100 children with a middle ear infection who took antibiotics, how many experienced an adverse effect (e.g., vomiting, diarrhoea or rash)?

[c_ear_f10_recall] {open-int min=0 max=100} Out of 100 children with a middle ear infection who took placebo, how many continued to have impaired hearing 4-6 weeks after diagnosis?

[c_ear_f2_recall] Children who took which treatment had a higher chance of developing a ruptured eardrum?

<1> Antibiotics

<2> Both antibiotics and placebo were the same

<3> Placebo

<4> This information is not shown

<5> I don't know

{end page ear1x_recall}

{page ear2x_recall}

[c_ear_f3_recall] Children who took which treatment had a higher chance of experiencing pain 4-7 days after diagnosis?

<1> Antibiotics

<2> Both antibiotics and placebo were the same

<3> Placebo

<4> This information is not shown

<5> I don't know

[c_ear_f4_recall] Out of 100 children who received placebo, how many had issues with hearing 4-6 weeks after diagnosis?

<1> Almost none

<2> About a quarter

<3> About half

<4> About three quarters

<5> I don't know

[c_ear_f12_recall] Which result was less common in children who took antibiotics compared with those who took a placebo?

<1> Pain

<2> Hearing issues

<3> Adverse effects

<4> This information is not shown

<5> I don't know

[c_ear_f5_recall] How did antibiotics affect children's hearing 4-6 weeks after diagnosis compared to placebo?

<1> Antibiotics REDUCED hearing

<2> Antibiotics IMPROVED hearing

<3> The effect was the same in both groups

<4> This information is not shown

<5> I don't know

{end page ear2x_recall}

{page ear3x_recall}

[c_ear_f6_recall] Which of these statements best describes the evidence shown here?

<1> Antiobiotics had no effect

<2> Antibiotics only caused harm

<3> Antibiotic only caused benefits

<4> Antibiotics caused both harm and benefits

<5> I don't know

[c_ear_f11_recall] Which group was less likely to experience pain 4-7 days after diagnosis?

<1> Children who took antibiotics

<2> Children who took a placebo

<3> The effect was the same in both groups

<4> This information was not shown

<5> I don't know

[c_ear_f7_recall] Which group experienced more adverse effects (harms) such as vomiting, diarrhoea or rash?

<1> Children who took antibiotics

<2> Children who took a placebo

<3> The effect was the same in both groups

<4> This information was not shown

<5> I don't know

[c_ear_f8_recall] {open-int min=0 max=100} How many more of the 100 children taking antibiotics experienced adverse effects (harms) compared to the 100 children taking placebo? Enter 0 if there was no difference.

{end page ear3x_recall}

{page explanation}

Thank you for answering those difficult questions. We were measuring how well people remembered the original information. Now, we will measure comprehension by showing you the evidence and asking you to answer the same questions for a final time.

{end page explanation}

{page earfactbox}

Assume you found the following information about antibiotics for treating inner ear infections in children. Please read the information to consider whether or not you would decide to choose antibiotic treatment for your child.<br><br>

The numbers below are for children 0-15 years of age with an acute middle ear infection who either received antibiotics or placebo (sugar pill) over a period of 7-14 days. <br><br>

Based on this information, please answer some questions. Do not rush: we will use the results of these studies to design messages for actual patients. Thank you for your help.<br><br>


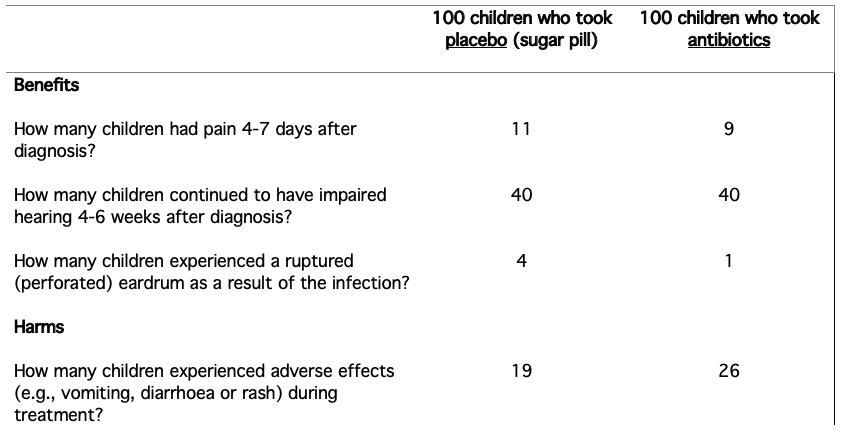


On the following pages, you will be shown this again and will answer questions about the content.


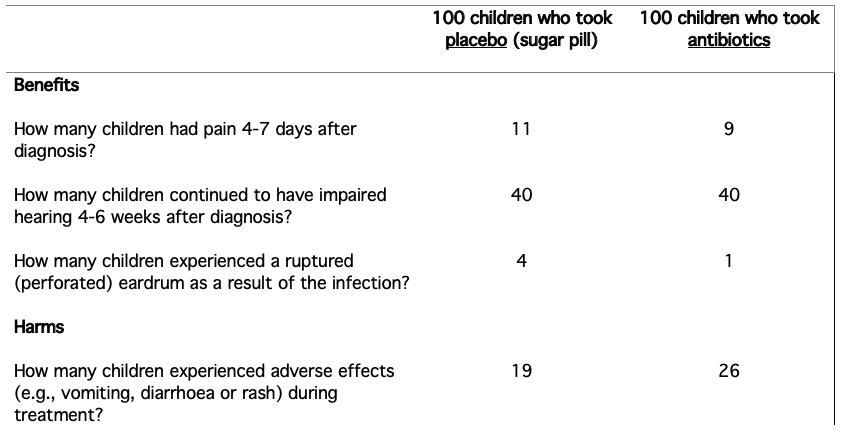


[c_ear_f1x] {open-int min=0 max=100} Out of 100 children with a middle ear infection who took antibiotics, how many experienced a ruptured eardrum?

[c_ear_f9x] {open-int min=0 max=100} Out of 100 children with a middle ear infection who took antibiotics, how many experienced an adverse effect (e.g., vomiting, diarrhoea or rash)?

[c_ear_f10x] {open-int min=0 max=100} Out of 100 children with a middle ear infection who took placebo, how many continued to have impaired hearing 4-6 weeks after diagnosis?

[c_ear_f2] Children who took which treatment had a higher chance of developing a ruptured eardrum?

<1> Antibiotics

<2> Both antibiotics and placebo were the same

<3> Placebo

<4> This information is not shown

<5> I don't know

{end page ear1x}

{page ear2x}


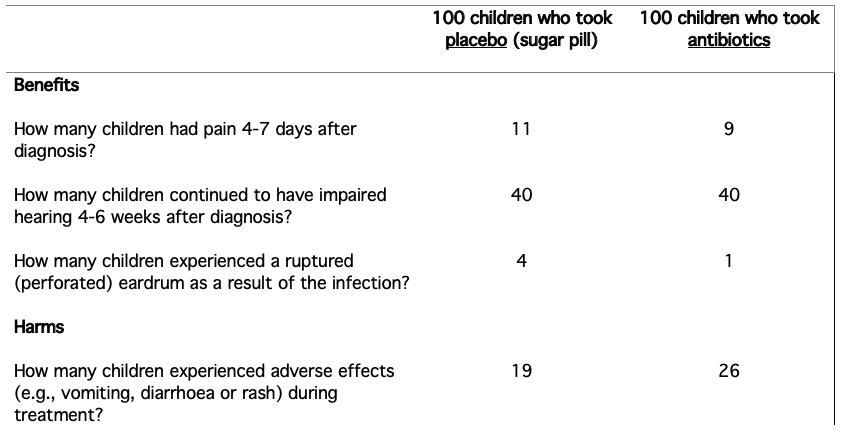


[c_ear_f3] Children who took which treatment had a higher chance of experiencing pain 4-7 days after diagnosis?

<1> Antibiotics

<2> Both antibiotics and placebo were the same

<3> Placebo

<4> This information is not shown

<5> I don't know

[c_ear_f4] Out of 100 children who received placebo, how many had issues with hearing 4-6 weeks after diagnosis?

<1> Almost none

<2> About a quarter

<3> About half

<4> About three quarters

<5> I don't know

[c_ear_f12] Which result was less common in children who took antibiotics compared with those who took a placebo?

<1> Pain

<2> Hearing issues

<3> Adverse effects

<4> This information is not shown

<5> I don't know

[c_ear_f5] How did antibiotics affect children's hearing 4-6 weeks after diagnosis compared to placebo?

<1> Antibiotics REDUCED hearing

<2> Antibiotics IMPROVED hearing

<3> The effect was the same in both groups

<4> This information is not shown

<5> I don't know

{end page ear2x}

{page ear3x}


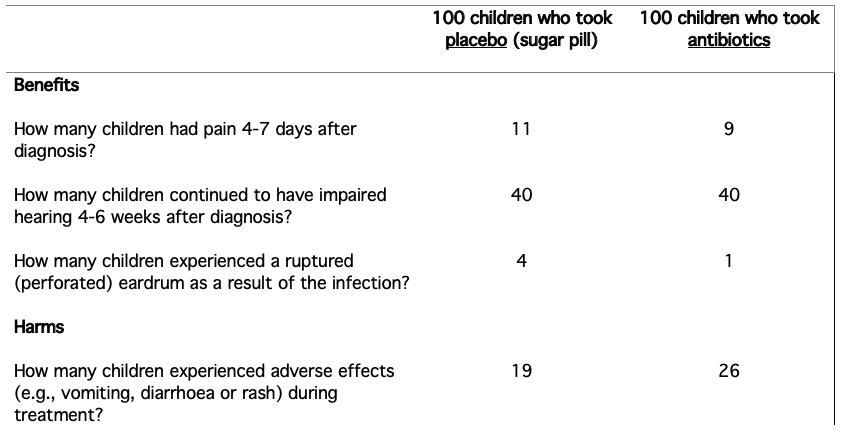


[c_ear_f6] Which of these statements best describes the evidence shown here?

<1> Antiobiotics had no effect

<2> Antibiotics only caused harm

<3> Antibiotic only caused benefits

<4> Antibiotics caused both harm and benefits

<5> I don't know

[c_ear_f11] Which group was less likely to experience pain 4-7 days after diagnosis?

<1> Children who took antibiotics

<2> Children who took a placebo

<3> The effect was the same in both groups

<4> This information was not shown

<5> I don't know

[c_ear_f7] Which group experienced more adverse effects (harms) such as vomiting, diarrhoea or rash?

<1> Children who took antibiotics

<2> Children who took a placebo

<3> The effect was the same in both groups

<4> This information was not shown

<5> I don't know

[c_ear_f8x] {open-int min=0 max=100} How many more of the 100 children taking antibiotics experienced adverse effects (harms) compared to the 100 children taking placebo? Enter 0 if there was no difference.

{end page ear3x}

[conflict_ear] {grid roworder=randomize} Now, thinking about the choice you just made and the information you read, please look at the following comments some people make when deciding about treatment. Please show how strongly you agree or disagree with these comments.

-[conflict_ear1] I know which options are available to me

-[conflict_ear2] I know the benefits of each option

-[conflict_ear3] I know the risks and side effects of each option

<1> Strongly disagree

<2> Disagree

<3> Somewhat disagree

<4> Neither agree nor disagree

<5> Somewhat agree

<6> Agree

<7> Strongly agree

{end module ear1}

{module ear2 if Cambridgesplit==2}


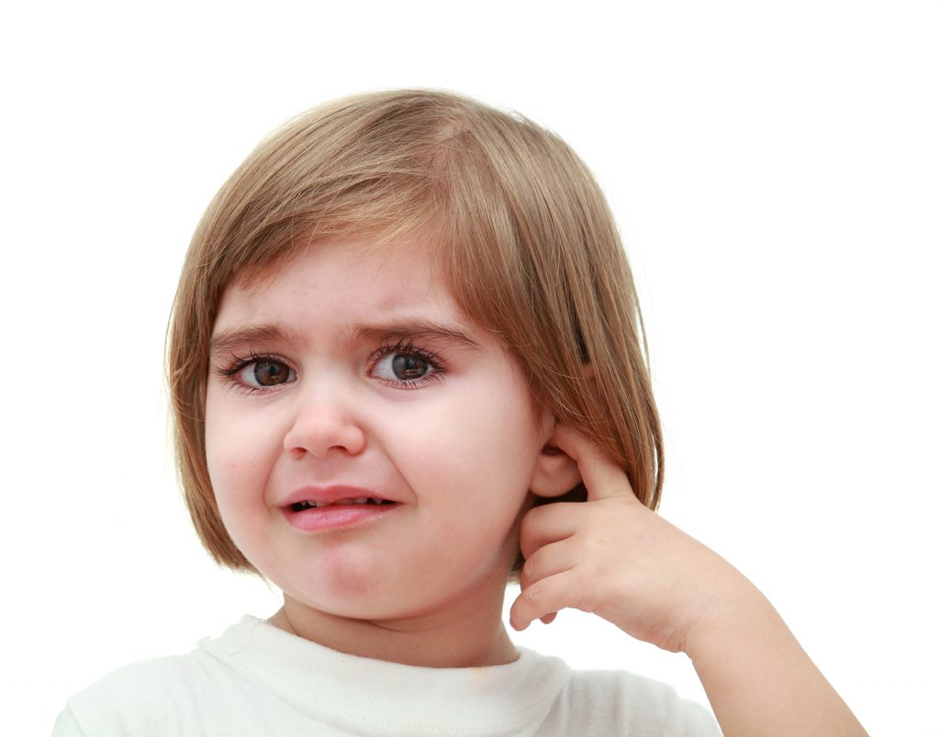


</br>

Ear infections are a common medical problem, especially in children, and can cause discomfort and hearing loss.<br><br>

[decision_eart] Imagine that a child you know is experiencing frequent ear infections. Based on the information you remember, please consider what decision you would personally make about them taking antibiotics.

<1> The child should take antibiotics

<2> The child should NOT take antibiotics

<3> It would not matter if they take antibiotics or not because the results are similar

<4> Unsure

{page earfactboxtext2_recall}

In the first part of the study a month ago, you saw information on antibiotics for children with ear infections. We provided you information on studies about whether antibiotics make ear infections clear up faster, or be less severe. The studies also looked at how many children experienced harms from the antibiotis such as vomiting, diarrhoea and rash. <br><br>

On the following pages we would like to ask you some questions about what you remember. The information from last month showed outcomes for a group who took antibiotics compared to those who took a placebo (a sugar pill). <br><br>

Please enter a guess for each answer, even if you don't remember. Please try for accuracy, but we don't expect you to remember all the details.

{end page earfactboxtext2_recall}

{page ear4x_recall}

[c_ear_t1_recall] {open-int min=0 max=100} Out of 100 children with a middle ear infection who took antibiotics, how many experienced a ruptured eardrum?

[c_ear_t9_recall] {open-int min=0 max=100} Out of 100 children with a middle ear infection who took antibiotics, how many experienced an adverse effect (e.g., vomiting, diarrhoea or rash)?

[c_ear_t10_recall] {open-int min=0 max=100} Out of 100 children with a middle ear infection who took placebo, how many continued to have impaired hearing 4-6 weeks after diagnosis?

[c_ear_t2_recall] Children who took which treatment had a higher chance of developing a ruptured eardrum?

<1> Antibiotics

<2> Both antibiotics and placebo were the same

<3> Placebo

<4> This information is not shown

<5> I don't know

{end page ear4x_recall}

{page ear5x_recall}

[c_ear_t3_recall] Children who took which treatment had a higher chance of experiencing pain 4-7 days after diagnosis?

<1> Antibiotics

<2> Both antibiotics and placebo were the same

<3> Placebo

<4> This information is not shown

<5> I don't know

[c_ear_t4_recall] Out of 100 children who received placebo, how many had issues with hearing 4-6 weeks after diagnosis?

<1> Almost none

<2> About a quarter

<3> About half

<4> About three quarters

<5> I don't know

[c_ear_t12_recall] Which result was less common in children who took antibiotics compared with those who took a placebo?

<1> Pain

<2> Hearing issues

<3> Adverse effects

<4> This information is not shown

<5> I don't know

[c_ear_t5_recall] How did antibiotics affect children's hearing 4-6 weeks after diagnosis compared to placebo?

<1> Antibiotics REDUCED hearing

<2> Antibiotics IMPROVED hearing

<3> The effect was the same in both groups

<4> This information is not shown

<5> I don't know

{end page ear5x_recall}

{page ear6x_recall}

[c_ear_t6_recall] Which of these statements best describes the evidence shown here?

<1> Antiobiotics had no effect

<2> Antibiotics only caused harm

<3> Antibiotic only caused benefits

<4> Antibiotics caused both harm and benefits

<5> I don't know

[c_ear_t11_recall] Which group was less likely to experience pain 4-7 days after diagnosis?

<1> Children who took antibiotics

<2> Children who took a placebo

<3> The effect was the same in both groups

<4> This information was not shown

<5> I don't know

[c_ear_t7_recall] Which group experienced more adverse effects (harms) such as vomiting, diarrhoea or rash?

<1> Children who took antibiotics

<2> Children who took a placebo

<3> The effect was the same in both groups

<4> This information was not shown

<5> I don't know

[c_ear_t8_recall] {open-int min=0 max=100} How many more of the 100 children taking antibiotics experienced adverse effects (harms) compared to the 100 children taking placebo? Enter 0 if there was no difference.

{end page ear6x_recall}

{page explanation2}

Thank you for answering those difficult questions. We were measuring how well people remembered the original information. Now, we will measure comprehension by showing you the evidence and asking you to answer the same questions for a final time.

{end page explanation2}

{page earfactboxtext2}

Assume you found the following information about antibiotics for treating inner ear infections in children. Please read the information to consider whether or not you would decide to choose antibiotic treatment for your child.<br><br>

The numbers below are for children 0-15 years of age with an acute middle ear infection who either received antibiotics or placebo (sugar pill) over a period of 7-14 days. <br><br>

Based on this information, please answer some questions. Do not rush: we will use the results of these studies to design messages for actual patients. Thank you for your help.<br><br>

Benefits: Of the 100 children who took antibiotics, 9 children had pain 4-7 days after diagnosis compared to 11 out of the 100 children who took the placebo (sugar pill). There was no difference between groups in how many children had impaired hearing 4-6 weeks after diagnosis (40 in each group). One out of 100 children who took antibiotics had a ruptured (perforated) eardrum as a result of the infection, compared to 4 out of 100 children who took placebo.<br><br>

Harms: 26 out of 100 children taking antibiotics experienced an adverse effect (e.g., vomiting, diarrhoea or rash), compared to 19 out of 100 children who took the placebo.<br><br>

On the following pages, you will be shown this again and will answer questions about the content.

</br>

{end page earfactboxtext2}

{page ear4x}

Benefits: Of the 100 children who took antibiotics, 9 children had pain 4-7 days after diagnosis compared to 11 out of the 100 children who took the placebo (sugar pill). There was no difference between groups in how many children had impaired hearing 4-6 weeks after diagnosis (40 in each group). One out of 100 children who took antibiotics had a ruptured (perforated) eardrum as a result of the infection, compared to 4 out of 100 children who took placebo.<br><br>

Harms: 26 out of 100 children taking antibiotics experienced an adverse effect (e.g., vomiting, diarrhoea or rash), compared to 19 out of 100 children who took the placebo.<br><br>

[c_ear_t1x] {open-int min=0 max=100} Out of 100 children with a middle ear infection who took antibiotics, how many experienced a ruptured eardrum?

[c_ear_t9x] {open-int min=0 max=100} Out of 100 children with a middle ear infection who took antibiotics, how many experienced an adverse effect (e.g., vomiting, diarrhoea or rash)?

[c_ear_t10x] {open-int min=0 max=100} Out of 100 children with a middle ear infection who took placebo, how many continued to have impaired hearing 4-6 weeks after diagnosis?

[c_ear_t2] Children who took which treatment had a higher chance of developing a ruptured eardrum?

<1> Antibiotics

<2> Both antibiotics and placebo were the same

<3> Placebo

<4> This information is not shown

<5> I don't know

{end page ear4x}

{page ear5x}

Benefits: Of the 100 children who took antibiotics, 9 children had pain 4-7 days after diagnosis compared to 11 out of the 100 children who took the placebo (sugar pill). There was no difference between groups in how many children had impaired hearing 4-6 weeks after diagnosis (40 in each group). One out of 100 children who took antibiotics had a ruptured (perforated) eardrum as a result of the infection, compared to 4 out of 100 children who took placebo.<br><br>

Harms: 26 out of 100 children taking antibiotics experienced an adverse effect (e.g., vomiting, diarrhoea or rash), compared to 19 out of 100 children who took the placebo.<br><br>

[c_ear_t3] Children who took which treatment had a higher chance of experiencing pain 4-7 days after diagnosis?

<1> Antibiotics

<2> Both antibiotics and placebo were the same

<3> Placebo

<4> This information is not shown

<5> I don't know

[c_ear_t4] Out of 100 children who received placebo, how many had issues with hearing 4-6 weeks after diagnosis?

<1> Almost none

<2> About a quarter

<3> About half

<4> About three quarters

<5> I don't know

[c_ear_t12] Which result was less common in children who took antibiotics compared with those who took a placebo?

<1> Pain

<2> Hearing issues

<3> Adverse effects

<4> This information is not shown

<5> I don't know

[c_ear_t5] How did antibiotics affect children's hearing 4-6 weeks after diagnosis compared to placebo?

<1> Antibiotics REDUCED hearing

<2> Antibiotics IMPROVED hearing

<3> The effect was the same in both groups

<4> This information is not shown

<5> I don't know

{end page ear5x}

{page ear6x}

Benefits: Of the 100 children who took antibiotics, 9 children had pain 4-7 days after diagnosis compared to 11 out of the 100 children who took the placebo (sugar pill). There was no difference between groups in how many children had impaired hearing 4-6 weeks after diagnosis (40 in each group). One out of 100 children who took antibiotics had a ruptured (perforated) eardrum as a result of the infection, compared to 4 out of 100 children who took placebo.<br><br>

Harms: 26 out of 100 children taking antibiotics experienced an adverse effect (e.g., vomiting, diarrhoea or rash), compared to 19 out of 100 children who took the placebo.<br><br>

[c_ear_t6] Which of these statements best describes the evidence shown here?

<1> Antiobiotics had no effect

<2> Antibiotics only caused harm

<3> Antibiotic only caused benefits

<4> Antibiotics caused both harm and benefits

<5> I don't know

[c_ear_t11] Which group was less likely to experience pain 4-7 days after diagnosis?

<1> Children who took antibiotics

<2> Children who took a placebo

<3> The effect was the same in both groups

<4> This information was not shown

<5> I don't know

[c_ear_t7] Which group experienced more adverse effects (harms) such as vomiting, diarrhoea or rash?

<1> Children who took antibiotics

<2> Children who took a placebo

<3> The effect was the same in both groups

<4> This information was not shown

<5> I don't know

[c_ear_t8x] {open-int min=0 max=100} How many more of the 100 children taking antibiotics experienced adverse effects (harms) compared to the 100 children taking placebo? Enter 0 if there was no difference.

{end page ear6x}

[conflict_eart] {grid roworder=randomize} Now, thinking about the choice you just made and the information you read, please look at the following comments some people make when deciding about treatment. Please show how strongly you agree or disagree with these comments.

-[conflict_eart1] I know which options are available to me

-[conflict_eart2] I know the benefits of each option

-[conflict_eart3] I know the risks and side effects of each option

<1> Strongly disagree

<2> Disagree

<3> Somewhat disagree

<4> Neither agree nor disagree

<5> Somewhat agree

<6> Agree

<7> Strongly agree

{end module ear2}

{module flu1 if Cambridgesplit==3}


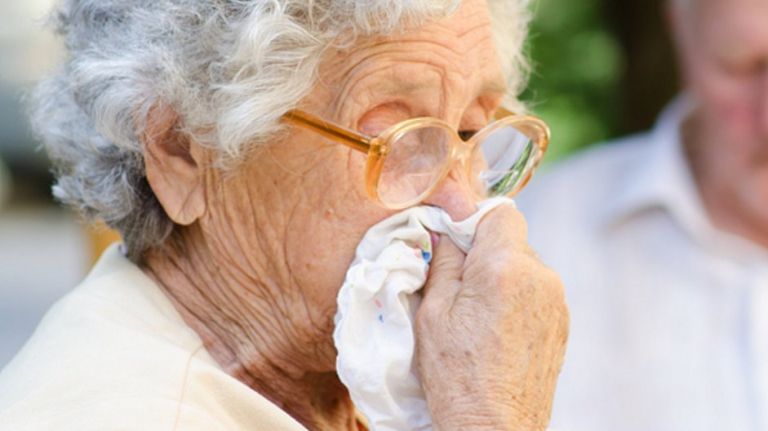


Influenza is a viral illness that is particularly dangerous in vulnerable populations such as children and the elderly, and can develop into serious illness.<br><br>

[decision_flu] Imagine that an older relative has been offered an influenza vaccination. Based on what you remember, please consider what decision you personally would make about them getting the vaccine.

<1> The older adult should get the influenza vaccine

<2> The older adult should NOT get the influenza vaccine

<3> It would not matter if they get the vaccine or not because the results are similar

<4> Unsure

{page flufactbox_recall}

In the first part of the study a month ago, you saw information on whether the influenza vaccine reduces the likelihood and severity of symptoms in people over 60. These studies also compared how many of these people experienced harms from the vaccine, such as pain, tenderness, or swelling at the injection site.<br><br>

On the following pages we would like to ask you some questions about what you remember. The information from last month showed outcomes for a group who took the influenza vaccine compared to those who took a placebo (a dummy injection - no vaccine).<br.<br>

Please enter a guess for each answer, even if you don't remember. Please try for accuracy, but we don't expect you to remember all the details.

{end page flufactbox_recall}

{page flu1x_recall}

[c_flu_f1_recall] {open-int min=0 max=1000} Out of 1,000 adults aged 60 or older who received the influenza vaccine, how many died from any cause within one year?

[c_flu_f9_recall] {open-int min=0 max=1000} Out of 1,000 adults aged 60 or older who received the influenza vaccine, how many experienced pain or tenderness in their arm?

[c_flu_f10_recall] {open-int min=0 max=1000} Out of 1,000 adults aged 60 or older who received the placebo, how many developed an influenza-like illness?

[c_flu_f2_recall] Older adults who took which treatment had a higher chance of dying within one year?

<1> Influenza vaccine

<2> Both influenza vaccine and placebo were the same

<3> Placebo

<4> This information is not shown

<5> I don't know

{end page flu1x_recall}

{page flu2x_recall}

[c_flu_f3_recall] Older adults who took which treatment had a higher chance of developing influenza in the following year?

<1> Influenza vaccine

<2> Both influenza vaccine and placebo were the same

<3> Placebo

<4> This information is not shown

<5> I don't know

[c_flu_f4_recall] {open-int min=0 max=1000} Out of 1,000 adults aged 60 or older who received placebo, how many experienced redness, swelling, or hardening at the injection site?

[c_flu_f12_recall] Which result was more common in adults who received the influenza vaccine compared with those who took a placebo?

<1> Confirmed influenza

<2> Dying of any cause

<3> Pain or tenderness

<4> This information is not shown

<5> I don't know

[c_flu_f5_recall] How did receiving the influenza vaccine affect how many older adults developed an influenza-like illness compared with placebo?

<1> The influenza vaccine REDUCED influenza-like illnesses

<2> The influenza vaccine INCREASED influenza-like illnesses

<3> The effect was the same in both groups

<4> This information is not shown

<5> I don't know

{end page flu2x_recall}

{page flu3x_recall}

[c_flu_f6_recall] Which of these statements best describes the evidence shown here?

<1> The influenza vaccine had no effect

<2> The influenza vaccine only caused harm

<3> The influenza vaccine only caused benefits

<4> The influenza vaccine caused both harm and benefits

<5> I don't know

[c_flu_f11_recall] Which group was less likely to develop confirmed influenza?

<1> Older adults who took the influenza vaccine

<2> Older adults who took a placebo

<3> The effect was the same in both groups

<4> This information was not shown

<5> I don't know

[c_flu_f7_recall] Which group experienced more harms (such as pain or tenderness in the arm)?

<1> Older adults who took the influenza vaccine

<2> Older adults who took a placebo

<3> The effect was the same in both groups

<4> This information was not shown

<5> I don't know

[c_flu_f8_recall] {open} How many more of the 1,000 older adults who received the influenza vaccine experienced redness, swelling, or hardening at the injection site compared to the 1,000 older adults who received a placebo?

{end page flu3x_recall}

{page explanation3}

Thank you for answering those difficult questions. We were measuring how well people remembered the original information. Now, we will measure comprehension by showing you the evidence and asking you to answer the same questions for a final time.

{end page explanation3}

{page flufactbox}

Assume you found the following information about vaccines to prevent influenza in older adults. Please read the information to consider whether or not you would decide to recommend a vaccine for an older relative.<br><br>

The numbers below are for adults aged 60 or older who were observed for one year. Older adults with placebo received an injection with a saline solution (no vaccine) instead of the influenza vaccine.

Based on this information, please answer some questions. Do not rush: we will use the results of these studies to design messages for actual patients. Thank you for your help.<br><br>


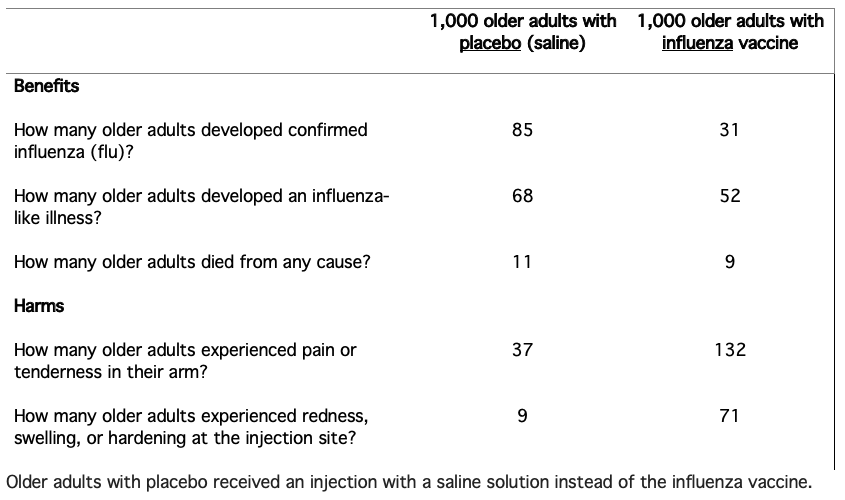


On the following pages, you will be shown this again and will answer questions about the content.

{end page flufactbox}

{page flu1x}


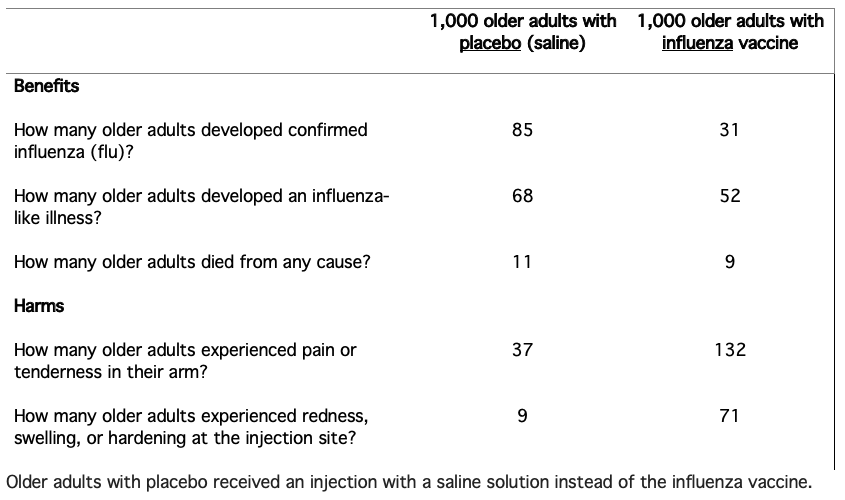


</br>

[c_flu_f1x] {open-int min=0 max=1000} Out of 1,000 adults aged 60 or older who received the influenza vaccine, how many died from any cause within one year?

[c_flu_f9x] {open-int min=0 max=1000} Out of 1,000 adults aged 60 or older who received the influenza vaccine, how many experienced pain or tenderness in their arm?

[c_flu_f10x] {open-int min=0 max=1000} Out of 1,000 adults aged 60 or older who received the placebo, how many developed an influenza-like illness?

[c_flu_f2] Older adults who took which treatment had a higher chance of dying within one year?

<1> Influenza vaccine

<2> Both influenza vaccine and placebo were the same

<3> Placebo

<4> This information is not shown

<5> I don't know

{end page flu1x}

{page flu2x}


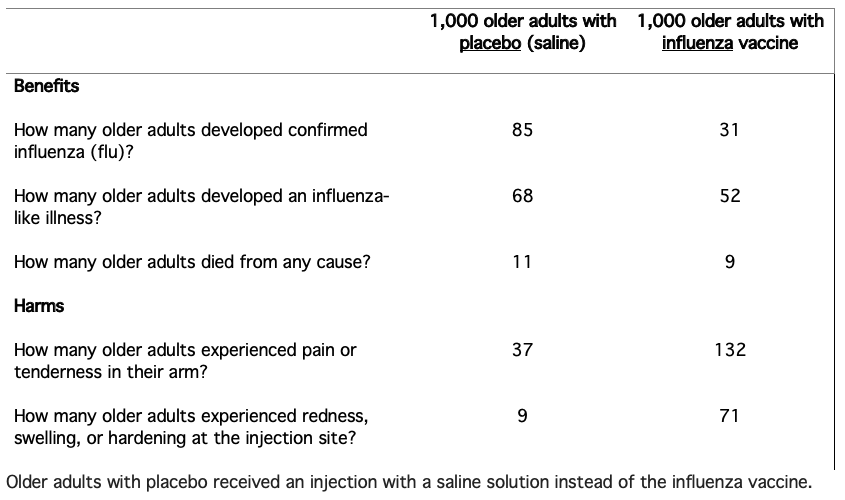


[c_flu_f3] Older adults who took which treatment had a higher chance of developing influenza in the following year?

<1> Influenza vaccine

<2> Both influenza vaccine and placebo were the same

<3> Placebo

<4> This information is not shown

<5> I don't know

[c_flu_f4x] {open-int min=0 max=1000} Out of 1,000 adults aged 60 or older who received placebo, how many experienced redness, swelling, or hardening at the injection site?

[c_flu_f12] Which result was more common in adults who received the influenza vaccine compared with those who took a placebo?

<1> Confirmed influenza

<2> Dying of any cause

<3> Pain or tenderness

<4> This information is not shown

<5> I don't know

[c_flu_f5] How did receiving the influenza vaccine affect how many older adults developed an influenza-like illness compared with placebo?

<1> The influenza vaccine REDUCED influenza-like illnesses

<2> The influenza vaccine INCREASED influenza-like illnesses

<3> The effect was the same in both groups

<4> This information is not shown

<5> I don't know

{end page flu2x}

{page flu3x}


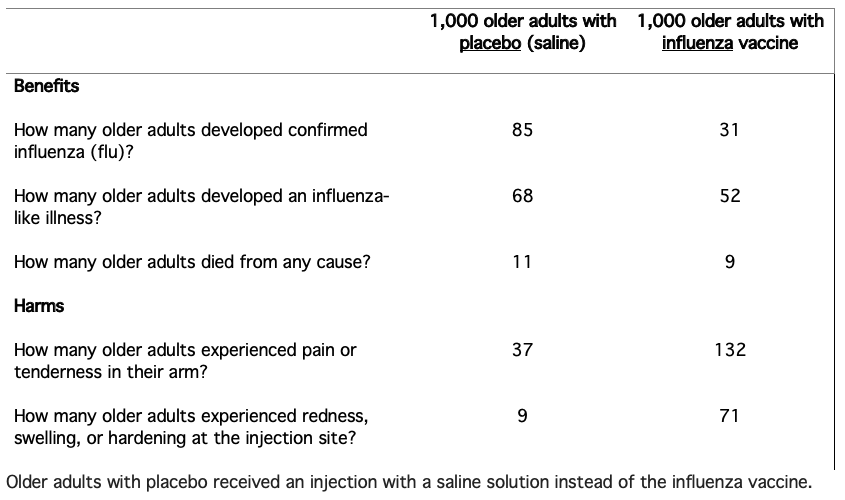


[c_flu_f6] Which of these statements best describes the evidence shown here?

<1> The influenza vaccine had no effect

<2> The influenza vaccine only caused harm

<3> The influenza vaccine only caused benefits

<4> The influenza vaccine caused both harm and benefits

<5> I don't know

[c_flu_f11] Which group was less likely to develop confirmed influenza?

<1> Older adults who took the influenza vaccine

<2> Older adults who took a placebo

<3> The effect was the same in both groups

<4> This information was not shown

<5> I don't know

[c_flu_f7] Which group experienced more harms (such as pain or tenderness in the arm)?

<1> Older adults who took the influenza vaccine

<2> Older adults who took a placebo

<3> The effect was the same in both groups

<4> This information was not shown

<5> I don't know

[c_flu_f8] {open} How many more of the 1,000 older adults who received the influenza vaccine experienced redness, swelling, or hardening at the injection site compared to the 1,000 older adults who received a placebo?

{end page flu3x}

[conflict_flu] {grid roworder=randomize} Now, thinking about the choice you just made and the information you read, please look at the following comments some people make when deciding about treatment. Please show how strongly you agree or disagree with these comments.

-[conflict_flu1] I know which options are available to me

-[conflict_flu2] I know the benefits of each option

-[conflict_flu3] I know the risks and side effects of each option

<1> Strongly disagree

<2> Disagree

<3> Somewhat disagree

<4> Neither agree nor disagree

<5> Somewhat agree

<6> Agree

<7> Strongly agree

{end moduleflu1}

{module flu2 if Cambridgesplit==4}


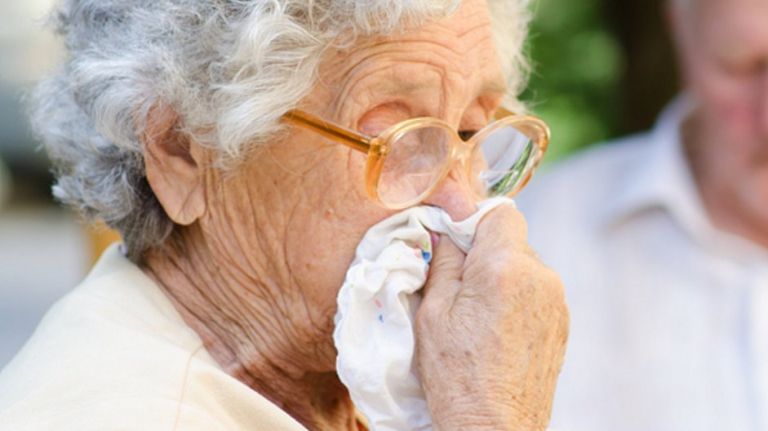


</br>

Influenza is a viral illness that is particularly dangerous in vulnerable populations such as children and the elderly, and can develop into serious illness.<br><br>

[decision_flu2] Imagine that an older relative has been offered an influenza vaccination. Based on what you remember, please consider what decision you personally would make about them getting the vaccine.

<1> The older adult should get the influenza vaccine

<2> The older adult should NOT get the influenza vaccine

<3> It would not matter if they get the vaccine or not because the results are similar

<4> Unsure

{page flufactbox2_recall}

In the first part of the study a month ago, you saw information on whether the influenza vaccine reduces the likelihood and severity of symptoms in people over 60. These studies also compared how many of these people experienced harms from the vaccine, such as pain, tenderness, or swelling at the injection site.<br><br>

On the following pages we would like to ask you some questions about what you remember. The information from last month showed outcomes for a group who took the influenza vaccine compared to those who took a placebo (a dummy injection - no vaccine).<br.<br>

Please enter a guess for each answer, even if you don't remember. Please try for accuracy, but we don't expect you to remember all the details.

{end page flufactbox2_recall}

{page flu4x_recall}

[c_flu_t1_recall] {open-int min=0 max=1000} Out of 1,000 adults aged 60 or older who received the influenza vaccine, how many died from any cause within one year?

[c_flu_t9_recall] {open-int min=0 max=1000} Out of 1,000 adults aged 60 or older who received the influenza vaccine, how many experienced pain or tenderness in their arm?

[c_flu_t10_recall] {open-int min=0 max=1000} Out of 1,000 adults aged 60 or older who received the placebo, how many developed an influenza-like illness?

[c_flu_t2_recall] Older adults who took which treatment had a higher chance of dying within one year?

<1> Influenza vaccine

<2> Both influenza vaccine and placebo were the same

<3> Placebo

<4> This information is not shown

<5> I don't know

{end page flu4x_recall}

{page flu5x_recall}

[c_flu_t3_recall] Older adults who took which treatment had a higher chance of developing influenza in the following year?

<1> Influenza vaccine

<2> Both influenza vaccine and placebo were the same

<3> Placebo

<4> This information is not shown

<5> I don't know

[c_flu_t4_recall] {open-int min=0 max=1000} Out of 1,000 adults aged 60 or older who received placebo, how many experienced redness, swelling, or hardening at the injection site?

[c_flu_t12_recall] Which result was more common in adults who received the influenza vaccine compared with those who took a placebo?

<1> Confirmed influenza

<2> Dying of any cause

<3> Pain or tenderness

<4> This information is not shown

<5> I don't know

[c_flu_t5_recall] How did receiving the influenza vaccine affect how many older adults developed an influenza-like illness compared with placebo?

<1> The influenza vaccine REDUCED influenza-like illnesses

<2> The influenza vaccine INCREASED influenza-like illnesses

<3> The effect was the same in both groups

<4> This information is not shown

<5> I don't know

{end page flu5x_recall}

{page flu6x_recall}

[c_flu_t6_recall] Which of these statements best describes the evidence shown here?

<1> The influenza vaccine had no effect

<2> The influenza vaccine only caused harm

<3> The influenza vaccine only caused benefits

<4> The influenza vaccine caused both harm and benefits

<5> I don't know

[c_flu_t11_recall] Which group was less likely to develop confirmed influenza?

<1> Older adults who took the influenza vaccine

<2> Older adults who took a placebo

<3> The effect was the same in both groups

<4> This information was not shown

<5> I don't know

[c_flu_t7_recall] Which group experienced more harms (such as pain or tenderness in the arm)?

<1> Older adults who took the influenza vaccine

<2> Older adults who took a placebo

<3> The effect was the same in both groups

<4> This information was not shown

<5> I don't know

[c_flu_t8_recall] {open-int min=0 max=1000} How many more of the 1,000 older adults who received the influenza vaccine experienced redness, swelling, or hardening at the injection site compared to the 1,000 older adults who received a placebo?

{end page flu6x_recall}

{page explanation4_recall}

Thank you for answering those difficult questions. We were measuring how well people remembered the original information. Now, we will measure comprehension by showing you the evidence and asking you to answer the same questions for a final time.

{end page explanation4_recall}

{page flufactbox2}

Assume you found the following information about vaccines to prevent influenza in older adults. Please read the information to consider whether or not you would decide to recommend a vaccine for an older relative.<br><br>

The numbers below are for adults aged 60 or older who were observed for one year. Older adults with placebo received an injection with a saline solution (no vaccine) instead of the influenza vaccine.<br><br>

Based on this information, please answer some questions. Do not rush: we will use the results of these studies to design messages for actual patients. Thank you for your help.<br><br>

Benefits: Of 1,000 adults ages 60 and older who received the influenza vaccine, 31 developed confirmed influenza (flu) over the next year, compared to 85 out of 1,000 older adults who received a placebo (no vaccine). Of older adults receiving the influenza vaccine, 52 out of 1,000 developed an influenza-like illness, while 68 out of 1,000 did in the placebo group. In the influenza vaccine group, 9 out of 1,000 died of all causes over the next year, while 11 out of 1,000 died in the placebo group.<br><br> Harms: Of the 1,000 older adults receiving the influenza vaccine, 132 experienced pain or tenderness in their arm, compared to 37 out of 1,000 in the placebo group. In the influenza vaccine group, 71 out of 1,000 experienced redness, swelling, or hardening at the injection site, compared to 9 out of 1,000 in the placebo group.<br><br><br>

On the following pages, you will be shown this again and will answer questions about the content.

{end page flufactbox2}

{page flu4x}

Benefits: Of 1,000 adults ages 60 and older who received the influenza vaccine, 31 developed confirmed influenza (flu) over the next year, compared to 85 out of 1,000 older adults who received a placebo (no vaccine). Of older adults receiving the influenza vaccine, 52 out of 1,000 developed an influenza-like illness, while 68 out of 1,000 did in the placebo group. In the influenza vaccine group, 9 out of 1,000 died of all causes over the next year, while 11 out of 1,000 died in the placebo group.<br><br> Harms: Of the 1,000 older adults receiving the influenza vaccine, 132 experienced pain or tenderness in their arm, compared to 37 out of 1,000 in the placebo group. In the influenza vaccine group, 71 out of 1,000 experienced redness, swelling, or hardening at the injection site, compared to 9 out of 1,000 in the placebo group.<br><br><br>

[c_flu_t1x] {open-int min=0 max=1000} Out of 1,000 adults aged 60 or older who received the influenza vaccine, how many died from any cause within one year?

[c_flu_t9x] {open-int min=0 max=1000} Out of 1,000 adults aged 60 or older who received the influenza vaccine, how many experienced pain or tenderness in their arm?

[c_flu_t10x] {open-int min=0 max=1000} Out of 1,000 adults aged 60 or older who received the placebo, how many developed an influenza-like illness?

[c_flu_t2] Older adults who took which treatment had a higher chance of dying within one year?

<1> Influenza vaccine

<2> Both influenza vaccine and placebo were the same

<3> Placebo

<4> This information is not shown

<5> I don't know

{end page flu4x}

{page flu5x}

Benefits: Of 1,000 adults ages 60 and older who received the influenza vaccine, 31 developed confirmed influenza (flu) over the next year, compared to 85 out of 1,000 older adults who received a placebo (no vaccine). Of older adults receiving the influenza vaccine, 52 out of 1,000 developed an influenza-like illness, while 68 out of 1,000 did in the placebo group. In the influenza vaccine group, 9 out of 1,000 died of all causes over the next year, while 11 out of 1,000 died in the placebo group.<br><br> Harms: Of the 1,000 older adults receiving the influenza vaccine, 132 experienced pain or tenderness in their arm, compared to 37 out of 1,000 in the placebo group. In the influenza vaccine group, 71 out of 1,000 experienced redness, swelling, or hardening at the injection site, compared to 9 out of 1,000 in the placebo group.<br><br><br>

[c_flu_t3] Older adults who took which treatment had a higher chance of developing influenza in the following year?

<1> Influenza vaccine

<2> Both influenza vaccine and placebo were the same

<3> Placebo

<4> This information is not shown

<5> I don't know

[c_flu_t4x] {open-int min=0 max=1000} Out of 1,000 adults aged 60 or older who received placebo, how many experienced redness, swelling, or hardening at the injection site?

[c_flu_t12] Which result was more common in adults who received the influenza vaccine compared with those who took a placebo?

<1> Confirmed influenza

<2> Dying of any cause

<3> Pain or tenderness

<4> This information is not shown

<5> I don't know

[c_flu_t5] How did receiving the influenza vaccine affect how many older adults developed an influenza-like illness compared with placebo?

<1> The influenza vaccine REDUCED influenza-like illnesses

<2> The influenza vaccine INCREASED influenza-like illnesses

<3> The effect was the same in both groups

<4> This information is not shown

<5> I don't know

{end page flu5x}

{page flu6x}

Benefits: Of 1,000 adults ages 60 and older who received the influenza vaccine, 31 developed confirmed influenza (flu) over the next year, compared to 85 out of 1,000 older adults who received a placebo (no vaccine). Of older adults receiving the influenza vaccine, 52 out of 1,000 developed an influenza-like illness, while 68 out of 1,000 did in the placebo group. In the influenza vaccine group, 9 out of 1,000 died of all causes over the next year, while 11 out of 1,000 died in the placebo group.<br><br> Harms: Of the 1,000 older adults receiving the influenza vaccine, 132 experienced pain or tenderness in their arm, compared to 37 out of 1,000 in the placebo group. In the influenza vaccine group, 71 out of 1,000 experienced redness, swelling, or hardening at the injection site, compared to 9 out of 1,000 in the placebo group.<br><br><br>

[c_flu_t6] Which of these statements best describes the evidence shown here?

<1> The influenza vaccine had no effect

<2> The influenza vaccine only caused harm

<3> The influenza vaccine only caused benefits

<4> The influenza vaccine caused both harm and benefits

<5> I don't know

[c_flu_t11] Which group was less likely to develop confirmed influenza?

<1> Older adults who took the influenza vaccine

<2> Older adults who took a placebo

<3> The effect was the same in both groups

<4> This information was not shown

<5> I don't know

[c_flu_t7] Which group experienced more harms (such as pain or tenderness in the arm)?

<1> Older adults who took the influenza vaccine

<2> Older adults who took a placebo

<3> The effect was the same in both groups

<4> This information was not shown

<5> I don't know

[c_flu_t8x] {open-int min=0 max=1000} How many more of the 1,000 older adults who received the influenza vaccine experienced redness, swelling, or hardening at the injection site compared to the 1,000 older adults who received a placebo?

{end page flu6x}

[conflict_flut] {grid roworder=randomize} Now, thinking about the choice you just made and the information you read, please look at the following comments some people make when deciding about treatment. Please show how strongly you agree or disagree with these comments.

-[conflict_flu1t] I know which options are available to me

-[conflict_flu2t] I know the benefits of each option

-[conflict_flu3t] I know the risks and side effects of each option

<1> Strongly disagree

<2> Disagree

<3> Somewhat disagree

<4> Neither agree nor disagree

<5> Somewhat agree

<6> Agree

<7> Strongly agree

{end moduleflu2}

[engagementx] {grid roworder=randomize} Next, recall the information you were shown about harms and benefits.

-[engagementx1] Would other people want to read this?

-[engagementx2] Are you interested in this information?

-[engagementx3] Do you like how this information is presented?

<1> Not at all

<2> A little

<3> Somewhat

<4> A fair amount

<5> Very much

[trust1] How reliable do you think these numbers are?

<1> 1 - Not at all

<2> 2

<3> 3

<4> 4

<5> 5 - Very reliable

[trust2] How trustworthy do you think these numbers are?

<1> 1 - Not at all

<2> 2

<3> 3

<4> 4

<5> 5 - Very trustworthy

[commentsx2] {open rows=15 cols=60 required=NONE} Did you have any problems with the survey? Any technical issues or problems with questions? Other comments for our team?

{page debrief}

Thank you for taking part in this study. This study is part of a project investigating how to communicate risks and evidence. The aim of the study is to understand how different ways of visualising and presenting data affect how easy they are to understand. <br><br> Some participants saw information about ear infection; others about influenza. Some participants saw tables of numbers; others read numbers written into normal sentences. The estimates are from real studies. However, we did not include information about how certain the numbers were, nor did we present variation across seasons (e.g. the flu vaccine may vary in its effectiveness across different flu seasons). The original influenza fact box shows ranges that represent averages across different seasons and different vaccines. These changes were made to make the experimental conditions more similar. The original fact boxes are here: https://www.harding-center.mpg.de/en/fact-boxes <br><br> If you have any questions or would like further information about the study, please email Dr Cameron Brick cb954@cam.ac.uk. If you would like to see the results, please let the researcher know and we will be happy to send you the information when the analyses are conducted. The study is now complete. Thank you for your time.

{end page debrief}
